# Glycine supplementation can partially restore oxidative stress-associated glutathione deficiency in ageing cats

**DOI:** 10.1017/S0007114524000370

**Published:** 2024-06-28

**Authors:** Avika Ruparell, Janet E. Alexander, Ryan Eyre, Laura Carvell-Miller, Y. Becca Leung, Samantha J. M. Evans, Lucy J. Holcombe, Martina Heer, Phillip Watson

**Affiliations:** 1 Waltham Petcare Science Institute, Melton Mowbray, Leicestershire, UK; 2 Royal Canin Pet Health and Nutrition Centre, 6574 State Route 503N, Lewisburg, OH, USA; 3 Royal Canin Research & Development Center, Aimargues, France; 4 The Ohio State University, 1925 Coffey Rd, Columbus, OH, USA

**Keywords:** Antioxidant, Feline, Senior, Amino acid

## Abstract

Intracellular levels of glutathione, the major mammalian antioxidant, are reported to decline with age in several species. To understand whether ageing affects circulating glutathione levels in cats, blood was sampled from two age groups, < 3 years and > 9 years. Further, to determine whether dietary supplementation with glutathione precursor glycine (GLY) affects glutathione concentrations in senior cats (> 8 years), a series of free GLY inclusion level dry diets were fed. Subsequently, a 16-week GLY feeding study was conducted in senior cats (> 7 years), measuring glutathione, and markers of oxidative stress. Whole blood and erythrocyte total, oxidised and reduced glutathione levels were significantly decreased in senior cats, compared with their younger counterparts (*P* ≤ 0·02). The inclusion level study identified 1·5 % free GLY for the subsequent dry diet feeding study. Significant increases in erythrocyte total and reduced glutathione were observed between senior cats fed supplemented and control diets at 4 weeks (*P* ≤ 0·03; maximum difference of 1·23 µM). Oxidative stress markers were also significantly different between groups at 8 (*P* = 0·004; difference of 0·68 nG/ml in 8-hydroxy-2'-deoxyguanosine) and 12 weeks (*P* ≤ 0·049; maximum difference of 0·62 nG/mG Cr in F_2_-isoprostane PGF_2*α*_). Senior cats have lower circulating glutathione levels compared with younger cats. Feeding senior cats a complete and balanced dry diet supplemented with 1·5 % free GLY for 12 weeks elevated initial erythrocyte glutathione and altered markers of oxidative stress. Dietary supplementation with free GLY provides a potential opportunity to restore age-associated reduction in glutathione in cats.

A key hallmark of ageing is increased oxidative stress^(1)^. This entails a progressive decline in the ability to manage levels of oxidative damage, which results in changes to cellular proteins, lipids and nucleic acids^(2)^. DNA and RNA damage, in particular, have been postulated to play a central role in age-related loss of physiological functions, including immunosenescence^(3)^. Age-related increases in oxidative stress have been associated with chronic inflammation, cardiovascular and neurodegenerative disease, osteoarthritis and type 2 diabetes in humans^(4–6)^ and companion animals, namely cats^(7,8)^ and dogs^(7–10)^.

The oxidative damage equilibrium is dependent on the neutralisation of generated reactive oxygen species, a process governed by the capacity of several antioxidant defence systems. The most abundant intracellular antioxidant in mammals is glutathione, a tripeptide of glutamate, cysteine (CYS) and glycine (GLY). Availability of glutathione is critical; it regulates the oxidation state of cells. In turn, the ability to upregulate glutathione synthesis in response to demands is hypothesised to be an important determinant of cell survival^(11)^. Evidence from several rodent^(12–14)^, dog^(15,16)^ and human^(17–21)^ studies suggests that glutathione levels in organs, erythrocytes and plasma decline with age.

Studies in aged humans and rodents have shown that dietary supplementation with the precursors of glutathione, CYS and/or GLY, can restore glutathione synthesis and reduce levels of oxidative stress. Sekhar *et.al.* ^(21)^ observed that significantly lower concentrations of erythrocyte glutathione in elderly human subjects, compared with younger control subjects, could be re-established by dietary GLY (1·33 mM/kg/d) and CYS (0·81 mM/kg/d) supplementation for 14 d. Not only was erythrocyte glutathione significantly increased, but plasma reactive oxygen metabolites, plasma F_2_-isoprostanes (F_2_-IsoPs) and lipid peroxides were significantly reduced. A recent intervention study by Kumar *et al.* ^(22)^ supplementing 100 mg/kg/d GLY and CYS, provided as N-acetylcysteine (100 mg/kg/d), improved glutathione deficiency, oxidative stress, mitochondrial dysfunction, inflammation and physical function. Perturbations to glutathione homoeostasis have also been explored in disease models. Linking HIV to glutathione deficiency, Nguyen *et al.* ^(23)^ explored supplementation with both glutathione precursors in infected male patients. The authors observed 2 weeks of oral supplementation of 1·33 mM/kg/d GLY and 0·81 mM/kg/d CYS restored glutathione synthesis, improved mitochondrial fat and carbohydrate oxidation, insulin sensitivity, body composition, muscle strength and dyslipidaemia.

Supplementation of GLY alone has been investigated in rodent models. In a sucrose-fed rat model, where animals developed significantly lower aortic tissue glutathione and higher aortic tissue oxidised glutathione (GSSG) compared with controls, 1 % v/v GLY offered in drinking water for 4 weeks restored glutathione levels in vascular tissue and significantly reduced markers of oxidative damage.^(24)^. Furthermore, GLY administration (1 g/kg for 21 d) was reported to attenuate the loss of fat and muscle mass and to reduce skeletal muscle inflammation in a mouse model of cancer cachexia^(25)^.

Oxidative stress and abnormal glutathione metabolism have been implicated in various feline diseases. Cats with chronic kidney disease have been shown to exhibit significantly lower reduced glutathione (GSH) and GSH to GSSG ratio and significantly higher GSSG compared with clinically normal, age-matched cats^(26)^. Lower concentrations of GSH have also been characterised in the liver tissue of cats with necroinflammatory liver disease and hepatic lipidosis compared with healthy cats^(27)^. Furthermore, a study of feline immunodeficiency virus infection by Webb *et al.* ^(28)^ identified significantly increased whole blood (WB) GSH and intracellular GSH concentrations in a reduced CD4 + T cell population. Despite these substantiations, knowledge of how WB or erythrocyte GSH, GSSG, GLY or CYS concentrations vary in cats of different ages is lacking. To elucidate this, we report a cross-sectional study to determine levels of total glutathione, GSH and GSSG in WB and erythrocytes, as well as plasma free GLY, CYS and methionine concentrations in young adult and senior cats offered a complete and balanced diet. Following the confirmation of reduced glutathione levels in senior cats, we explored the effect of free GLY supplementation in a complete and balanced dry diet, containing excess CYS, via two additional studies. Supplementation of GLY alone was selected as opposed to GLY and CYS since CYS can be toxic^(29)^. The impact of the intervention was determined by measuring the resulting erythrocyte and leucocyte glutathione concentrations, and biomarkers of oxidative stress.

## Experimental methods

### Animals

Domestic short haired cats (all neutered) were housed at the Pet Health and Nutrition Center (PHNC), Lewisburg, OH, USA, and all procedures were approved by the WALTHAM Animal Welfare and Ethical Review Body and the PHNC Institutional Animal Care and Use Committee. Cats were deemed healthy by a veterinarian at the start of each study with no evidence of systemic disease requiring treatment, for example, arthritis, diabetes, thyroid disorder, liver or renal impairment, and had not been vaccinated or prescribed any medication within 2 weeks of blood sampling. Routine housing, husbandry and exercise regimens were maintained throughout the course of each study. Cats were group housed in a free-living environment with indoor/outdoor access during the day (weather permitting) except twice daily for a period of 30 min, when they were individually housed for feeding. Rooms were fitted with environmental enrichment, and all cats had daily social human interactions, which included grooming and play with toys for a minimum of 20 min. Water was provided *ad libitum* at all times. The general health and overall condition of each animal were monitored daily by the animal care staff.

### Impact of ageing on glutathione (cross-sectional study)

The first study consisted of thirty-two healthy adult cats (twenty-two female, ten male) that took part in a cross-sectional study. Sixteen were classified as the ‘young’ group (twelve female, four male), with a median age of 2·9 years (range 1·3–2·9 years) and 16 as the ‘senior’ group (ten female, six male), with a median age of 9·7 years (range 9·2–13·2 years). Cats in both age groups were fed a commercially available complete and balanced dry diet (IAMS Multi-Cat, Mars Petcare) meeting the Association of American Feed Control Officials (AAFCO) nutrient profile for adult cats throughout the study. All animals were within 10 % of ideal body condition score (BCS), and diets were provided at levels to maintain ideal BCS throughout the study. Ideal BCS was determined using the Size, Health And Physical Evaluation (S.H.A.P.E.) system^(30)^. Maintenance energy requirements were calculated using an average energetic intake estimated for each cat based on individual feed intakes required to maintain an ideal BCS. Two 3-ml blood samples were collected from the medial saphenous vein at an interval of 1 month to compare WB and erythrocyte total glutathione, GSH and GSSG and free plasma CYS, GLY and methionine concentration.

### Comparing different levels of glycine supplementation (glycine inclusion level study)

**Fig. 1.**
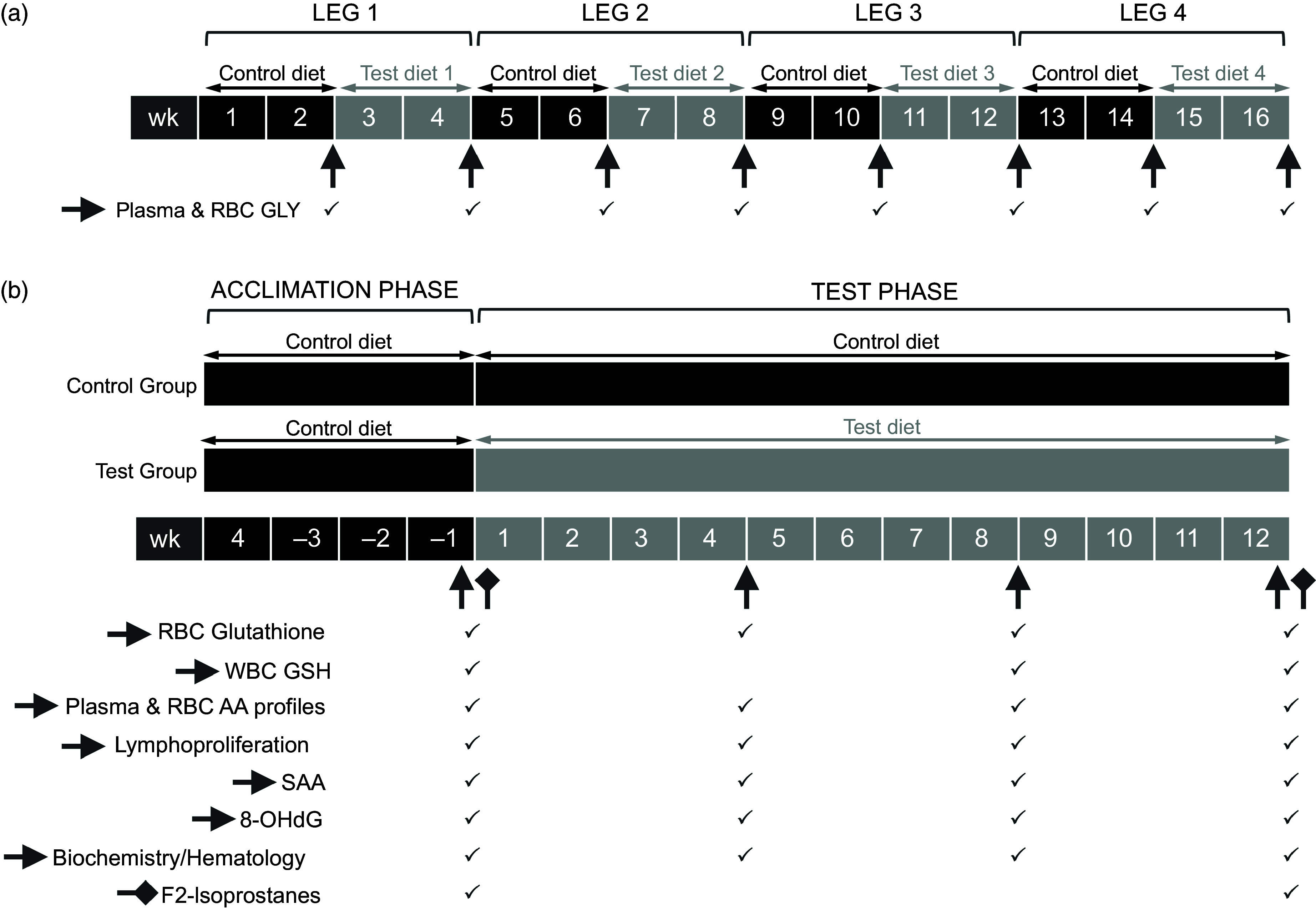
Glycine (a) inclusion level and (b) feeding study design schematics. Blood (triangular arrows) and urine (diamond arrows) sample collection time points and associated sample measures (ticked where measured).

To determine the level of GLY required to increase GLY concentrations in blood, fifty-two senior cats (thirty-three female, nineteen male), with a median age of 12·1 years (range 8·1–13·6 years), took part in a 16-week study, comprising diet rotations over eight, 2-week blocks (Fig. 1(a)). Cats were assigned to one of four groups and alternated between a control dry diet (IAMS Adult Cat Original Chicken, Mars Petcare) or test dry diet supplemented with either 0·5, 1·5 or 6·0 % as-fed free GLY. All diets were manufactured from the same batch of ingredients to the same formulation as commercially available diet. For test diets, GLY was added, as a dry powder ingredient, to the dry feed mix prior to extrusion. All diets were analysed for nutrient composition (online Supplementary Table 1) (total amino acid profiling method reference: AOAC 982·30 mod/AOAC 994·12 mod/AOAC 988·15; free amino acid profiling method reference: by performic acid oxidation followed by HPLC according to AOAC 994·12 mod and AOAC; Eurofins Nutrition Analysis Center), confirming target-free GLY supplementation levels had been achieved and that the diets met the AAFCO nutrient profile for adult cats. At the start of the study, all cats began on the control diet and were fed this diet for 2 weeks as an acclimation phase. Following this, cats were subsequently fed each test diet in a randomised order for 2 weeks, discriminated by room location, with a 2-week washout with the control diet in between. The animals were within 10 % of ideal BCS, and the diets provided were at levels to maintain ideal BCS throughout the study. Each 2-week test block concluded with a 4 ml fasted (overnight ≥ 18 h) blood sample collected from the medial saphenous vein from each cat into a sodium heparin vacutainer (BD Vacutainer®). Heparin-anticoagulated WB was processed to determine plasma and erythrocyte-free amino acid (AA) profiles, of which GLY in both components formed the primary measure. Blood samples collected at the end of a washout block represented the baseline sample for the subsequent 2-week test feeding block. Blood samples collected at the end of a test block represented the final, end point sample for the preceding 2-week block of feeding test diet.

### Impact of glycine supplementation on glutathione and markers of oxidative stress (glycine feeding study)

Forty-four senior cats (twenty-eight female, sixteen male), of which thirty-nine participated in the GLY inclusion level study, with a median age of 11·7 years (range 7·7–13·8 years), took part in the 16-week study, comprising a 4-week acclimation phase and a 12-week test phase (Fig. 1(b)). For the 4-week duration of the acclimation phase, cats were offered a control dry diet (IAMS Adult Cat Original Chicken, Mars Petcare). Subsequently, for the test phase, the senior cohort was randomly divided into two groups of twenty-two cats, one group remained on the control dry diet and the other was immediately transferred to a test dry diet supplemented with 1·5 % as-fed free GLY. The control group (fourteen female, eight male) had a mean age of 11·4 (sd 1·9) years while the test group (fourteen female, eight male) had a mean age of 11·5 (sd 2) years. Diets fed were analysed for nutrient composition and met the AAFCO nutrient profile for adult cats (online Supplementary Table 1). Total GLY and CYS levels were 2·42 % and 0·41 % in the control diet, and 3·94 % and 0·42 % in the test diet, respectively (online Supplementary Table 1). Blood and urine were collected at the end of the pre-test phase (from cats fasted overnight ≥ 18 h) to act as a baseline for all measures. Blood was collected from the medial saphenous vein for the measurement of erythrocyte glutathione profiles, leucocyte GSH, plasma and erythrocyte AA profiles, mitogen-induced lymphoproliferative response, serum amyloid A (SAA), 8-hydroxy-2'-deoxyguanosine (8-OHdG), one of the major products of DNA oxidation widely used as a biomarker of oxidative stress^(31)^, and biochemistry and haematology parameters (Fig. 1(b)). Urine was collected in lodges, to which the cats had been habituated, for the measurement of F_2_-IsoPs for additional quantification of oxidative damage. Subsequently, blood was collected every 4 weeks to mirror the measurement of parameters conducted at the end of the acclimation phase, with the exception of leucocyte glutathione being omitted at week 4. Subsequent urine collection was conducted at the end of the test phase and study (week 12). Aliquots for the majority of the blood-based measures were obtained from 8 ml heparin-anticoagulated WB (BD Vacutainer® 4 ml Heparin Tubes) with the exception of samples for biochemistry (1·2 ml; serum separator tube, SST, SARSTEDT, Inc.), haematology (1 ml; EDTA, BD Vacutainer®), SAA (1·2 ml; SST, SARSTEDT, Inc.) and leucocyte glutathione (1 ml; EDTA, BD Vacutainer®), which were collected into individual blood tubes.

### Sample analysis

#### Erythrocyte glutathione measurement

For the study evaluating the impact of ageing on glutathione, heparin-anticoagulated WB was placed on a rocker for at least 1 min before a 2 ml aliquot was removed for amino acid analysis. The remaining 1 ml sample was centrifuged at 1000 × ***g*** for 30 min at 4°C, and the buffy coat was then removed from the erythrocyte pellet. Determination of GSH and GSSG was carried out by enzymatic recycling of GSSG to GSH using a glutathione detection ELISA according to manufacturer’s instructions (Enzo Life Sciences, Farmingdale, Cat No. ADI-900–160) including pre-treatment of samples with metaphosphoric acid to remove interfering proteins. Absorbance measurements were made at 405 nm for 12 min at 1 min intervals with a microplate reader using kinetic mode (Cytation 3 multi-mode, BioTek). WB and erythrocyte total glutathione, GSH, GSSG and their ratio were determined; GSH was calculated as the difference between total glutathione and GSSH. Data were not normalised to Hb based on the assumption that variation in erythrocyte Hb levels in healthy cats is minimal.

In the GLY supplementation study, 1 ml heparin-anticoagulated WB was aliquoted and centrifuged at 2000 × ***g*** for 10 min. The buffy coat was then removed from the erythrocyte pellet and the pellet washed with PBS three times. The pellet was then flushed with nitrogen gas, snap frozen in liquid N_2_ and stored at −80°C. Samples were shipped on dry ice to Creative Proteomics for the quantification of erythrocyte glutathione. An internal standard (IS) solution, containing 13C isotope-labelled GSH and 13C isotope-labelled GSSG, was prepared in an antioxidation buffer. Serially diluted IS solutions were also prepared in the antioxidation buffer. Erythrocyte pellets were thawed on ice and diluted 50× with the antioxidation buffer. Fifty microlitres of each erythrocyte solution or IS calibration solution was mixed with 50 µl IS solution and 300 µl 40-mM N-ethylmaleimide-acetonitrile. After vortex-mixing for 10 min at room temperature, the resulting solutions were centrifuged, and the supernatant diluted 5× with water. Ten-microlitre aliquots of the diluted supernatant were analysed by the UPLC-MRM MS on an Agilent 1290 UHPLC system coupled to an Agilent 6495B QQQ mass spectrometer with positive-ion detection. Separation was carried out using a 10 cm long C18 UPLC column with an ammonium acetate buffer (A) and methanol (B) as the mobile phase for gradient elution (5–75 % B in 10 min), at 40 °C and 0·20 ml/min. Concentrations of GSH and GSSG were calculated from the IS calibration by interpolating the constructed linear-regression curves, with the analyte to IS peak ratios measured from sample solutions.

#### Plasma and erythrocyte free amino acid quantification

Heparin-anticoagulated WB was centrifuged at 4 (sd 2)°C for 30 min at 3000 ***g*** (cross-sectional study) and 10 min at 2000 ***g*** (GLY inclusion level and supplementation studies) to separate the plasma, buffy coat and erythrocytes. For the cross-sectional study, plasma was removed and deproteinised with 6 % sulfosalicylic acid (1:1) to remove plasma proteins and enable reliable determination of free plasma CYS^(32)^. Samples were centrifuged at 4000 ***g*** for 25 min. The supernatant was filtered through a 0·45 mm PTFE filter, pH adjusted to 2·2 and frozen immediately at –80°C. For the GLY inclusion level and supplementation studies, plasma was removed and frozen immediately at –80°C. The erythrocyte pellet was washed three times in cold PBS before also being stored at –80°C. Complete free amino acid determination of plasma and erythrocytes was performed at the Amino Acid Laboratory, University of California (Davis), USA^(33)^. Amino acid quantification was conducted using a Biochrom 30 amino acid analyser (Biochrom Ltd.).

#### Leucocyte glutathione measurement

Measurement of GSH concentration in leucocyte subsets was performed by The Ohio State University Veterinary Clinical Flow Cytometry Service (Columbus, OH, USA). Methods performed were adapted from Webb *et al.* ^(34)^ utilising a Cytek^®^ Northern Lights (Cytek Biosciences Inc.) spectral flow cytometer. Markers of leucocyte subsets CD21 (B-cells; clone CA2.1D6, Bio-Rad Laboratories Inc., Cat No. MCA1781R), CD4 (helper T-cells; clone vpg34, Bio-Rad Laboratories Inc., Cat No. MCA1346F), CD8 (cytotoxic T-cells; clone vpg9, Bio-Rad Laboratories Inc., Cat No. MCA1347GA), CD14 (monocytes, clone TÜK4, Bio-Rad Laboratories Inc., Cat No. MCA1568GA) and a viability marker (propidium iodide, Sigma-Aldrich, Merck, Cat No. P4170) were used to segregate leucocyte subsets as part of a single multiplexed panel. In addition to the above subsets, granulocytes (neutrophils, eosinophils and basophils together) were quantified and segregated according to characteristic light scatter properties and the absences of expression of CD21, CD4, CD8 or CD14. Briefly, to assess GSH in the cells, a non-fluorescent substrate, monochlorobimane (mBCl, Thermo Fisher Scientific Inc., Cat No. M1381MP), was included in the panel, which forms fluorescent adducts with glutathione when catalysed by the enzyme glutathione-S-transferase. The median florescence intensity of the mBCl–glutathione complex was recorded for each leucocyte population subset (B-cell, CD4 + T-cells, CD8 + T-cells, monocytes and granulocytes). The median florescence intensity was then correlated with the amount of GSH per cell using a standard curve generated by serial dilutions of known quantities of feline peripheral blood leucocytes assessed by both flow cytometry and a commercial glutathione assay kit (Cayman Chemical). Pairwise contrasts were performed for each of the leucocyte subsets considered both as the number of each subset per µl of blood (absolute cell counts) and as the amount of GSH per cell within each subset.

#### Mitogen-induced lymphoproliferative response

Heparin-anticoagulated WB was centrifuged at room temperature for 30 min at 390 ***g***. The buffy coat was removed and leucocytes isolated by Histopaque gradient density separation (Histopaque^®^-1077, Merck, Cat No. 10771 and Histopaque^®^-1119, Merck Cat No. 11191). Isolated leucocytes were counted using a Beckman Z2 particle cell counter, plated at 1 × 10^6^ cells/well in triplicate for each stimulation condition. Concanavalin A (Con A; from Jack Bean, Merck, Cat No. C5275) was added to the wells at either 0, 1 or 10 mg/ml. Cells were incubated at 37°C in a 5 % CO_2_ enriched environment for 96 h. After incubation, plates were centrifuged at room temperature for 10 min at 350 ***g*** and a tetrazolium dye MTT (3-(4,5-dimethylthiazol-2-yl)-2,5-diphenyltetrazolium bromide) assay (Merck, Cat No. M5655) performed as per manufacturer’s instructions to quantify viable cells as a proxy of proliferation relative to the control.

#### Serum amyloid A

It was measured using the Multispecies ‘PHASE’™ Serum Amyloid A ELISA Assay Kit (TriDelta Development Ltd., Cat No. TP-802) according to the manufacturer’s instructions.

#### Oxidative DNA damage

8-OHdG was measured using the OxiSelect™ Oxidative DNA Damage ELISA Kit (Cell Biolabs, Inc., Cat No. STA-320) according to the manufacturer’s instructions. Urine samples collected for the measurement of F_2_-IsoPs were stored at –80°C prior to shipment to Vanderbilt Eicosanoid Core Laboratory (Nashville, Tennessee, USA) for quantification of F_2_-IsoPs F_2*α*_ (PGF_2*α*_), free 8-iso-prostaglandin F_2*α*_ (8-iso-PGF_2*α*_), 2,3-dinor-5,6-dihydro-15-F_2t_-IsoP and 5-series F_2_-IsoP by GC-negative ion chemical ionisation-MS employing stable isotope dilution as described in Milne *et al.* ^(35)^ and Milne *et al.* ^(36)^.

#### Biochemistry and haematology

These were performed at IDEXX laboratories, USA. See online Supplementary Table 4 and 5 for biochemistry and haematology parameters, respectively.

### Statistical methods

The sample size for the cross-sectional study was determined using variance estimates for erythrocyte total glutathione from a previous unpublished study in dogs at the Waltham Petcare Science Institute. In order to use these data to power for this cross-sectional study, variability in cats was assumed to be comparable to dogs. The variance components were used to simulate 1000 data sets, with the 25 % effect size in the senior group. A linear mixed effects model was fit to each data set, with age as the fixed effect and animal as the random effect. Planned comparisons for this study were between the young adult and senior groups.

Data on GLY from a previous, unpublished study conducted in cats were used to estimate the variance components for calculating the sample size for the GLY inclusion level study. The variance components for plasma GLY and erythrocyte GLY were estimated and used to simulate 1000 data sets, with fold changes of 23 % and 30 % induced on the final sampling occasion in one diet group for plasma GLY and erythrocyte GLY, respectively. A linear mixed effects model was fit to each data set for each measure with diet, sampling occasion and their interaction as the fixed effects and individual cat as the random variable. Planned comparisons for the GLY inclusion level study were the change from baseline at each time point compared between groups.

Data collated from the cross-sectional study were used to power for the GLY feeding study by estimating the variance components of erythrocyte total glutathione in pmoles and simulating 1000 data sets, with a 40 %-fold change induced on final sampling occasion. A linear mixed effects model was fit to each data set, for each measure, with diet group, sample occasion and their interaction as the fixed effects and individual cat as the random effect. Planned comparisons for the GLY feeding study were between the diet groups at each sampling occasion.

For all analyses, power was calculated as the percentage of simulated data sets where all planned contrasts involving the fixed effect level where the effect size was induced were statistically significant. The sample size was determined as the minimum number of animals required to detect the desired effect size with a minimum of 80 % power.

For the cross-sectional study, all sampling occasions were combined prior to being fit to a linear mixed effects model, with age group as the fixed effects and individual cat as the random effects. The model residuals were examined visually to assess the assumptions of the model (linearity of predictors, normality of residuals, independence of variables and homoscedasticity). To improve agreement with model assumptions, a log10 transformation was applied to some parameters, prior to model fitting. Plasma GSH to GSSG ratio data was log10 transformed after the addition of 1 (the minimum value to remove negative values). The mean of each measure for each age group and the difference between age groups (or fold change where data were log10 transformed) were estimated with 95 %CI.

For all measures in the GLY inclusion level and feeding studies, the data were fit to a linear mixed effects model (to account for repeated measures), with diet, sampling occasion and their interaction as the fixed effects, and individual cat as the random effect. The model residuals were examined visually to assess the assumptions of the model. To improve agreement with model assumptions, a log10 transformation was applied to some parameters, prior to model fitting. The estimated means and 95 % family-wise CI were extracted from the model for each diet at each sampling occasion.

In the GLY inclusion level study, the change from baseline to each subsequent time point was compared between the diets.

With the data from the GLY feeding study, the baseline value for each animal was included in the model as a covariate to account for any differences between the groups that may exist. Contrasts were made between diets at each of the sampling occasions.

For both the cross-sectional and GLY feeding studies, the primary measure was erythrocyte total glutathione, while the primary variables for the GLY inclusion level study were plasma and erythrocyte GLY. For all comparisons, the estimated differences or fold changes were reported alongside the 95 % family-wise CI and single-step adjusted *P*-values. Statistically significant differences were determined when the *P*-value ≤ 0·05 for all measures. Statistical analyses were performed in R v4.1.2^(37)^ using libraries *nlme* ^(38)^, *multcomp* ^(39)^, *lme4* and *ggplot2* ^(40)^.

## Results

### Impact of ageing on glutathione (cross-sectional study)

Of the thirty-two cats enrolled for the cross-sectional study, one of the young adult cats, aged 2·8 years, was removed for health reasons unrelated to the study prior to any data being recorded. In addition, three cats (two young and one senior) had one measure missing for WB glutathione due to not obtaining sufficient blood during sampling.

#### Erythrocyte and whole blood glutathione

**Fig. 2.**
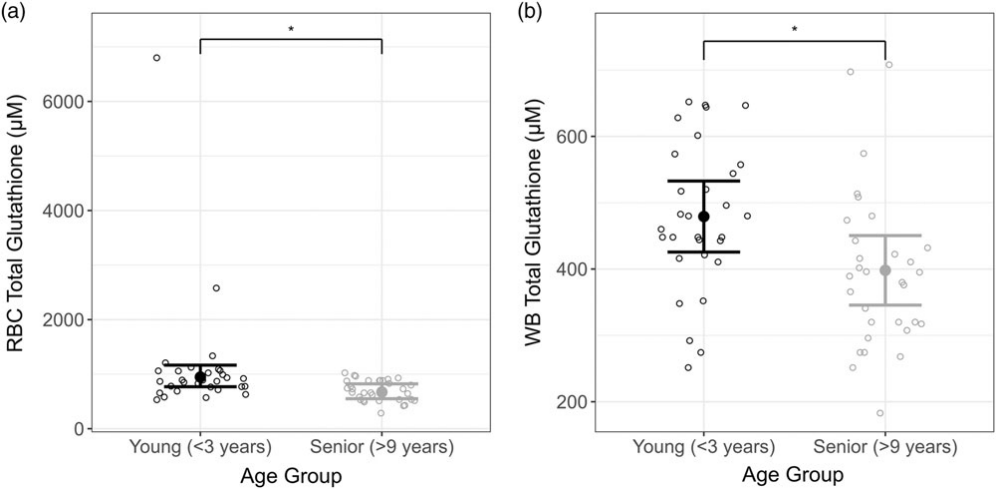
Mean (a) erythrocyte and (b) whole blood (WB) total glutathione (µM) by age group. Individual data are shown as open circles and means as solid circles with 95 % CI. * indicates significance between groups (*P* ≤ 0·05).

Significantly lower erythrocyte total glutathione was observed in the senior cats (> 9 years) compared with the young adult (< 3 years) age group (fold change 0·7, 95 % CI 0·6, 0·9; *P* = 0·008) (Fig. 2(a), Table 1). Mean erythrocyte total glutathione was 672·3 (95 % CI 549·8, 821·9) µM in the senior cats and 946·6 (95 % CI 769·1, 1165) µM in the younger adult group. Additionally, significantly lower WB total glutathione was observed in the senior compared with the young adult age group (difference –4·1, 95 % CI –7·3, –0·8 µM; *P* < 0·02) (Fig. 2(b), Table 1). Furthermore, lower concentrations of GSH (erythrocyte: fold change 0·4, 95 % CI 0·2, 0·6; WB: difference –6·9, 95 % CI –10·6, –3·2 µM) and higher concentrations of GSSG (erythrocyte: fold change 3·8, 95 % CI 2·5, 5·7; WB: fold change 2·8, 95 % CI 1·9, 4·2) were observed in the senior animals, leading to further significant differences compared with the younger adult cohort in erythrocyte and WB (all *P* < 0·001) (Table 1).

**Table 1.** Erythrocyte and whole blood (WB) glutathione concentrations, and free plasma glycine (GLY), cysteine (CYS) and methionine levels in the cross-sectional study young adult and senior cats

Measure	Young adult (< 3 years)		Senior (> 9 years)		Difference (senior – young adult) in means		Fold change (senior/young adult) in means		*P*
	Mean	95 % CI	Mean	95 % CI	Mean	95 % CI	Mean	95 % CI	
Erythrocyte total glutathione (µM)	946·6	769·1, 1165	672·3	549·8, 821·9			0·7	0·6, 0·9	0·008
Erythrocyte GSH (µM)	858·3	586·5, 1256·1	302·7	209·4, 437·8			0·4	0·2, 0·6	< 0·001
Erythrocyte GSSG (µM)	71·4	51·1, 99·7	268	193·9, 370·3			3·8	2·5, 5·7	< 0·001
Erythrocyte GSH:GSSG	12	6·9, 21·1	1·12	0·65, 1·93			0·1	0·1, 0·2	< 0·001
WB total glutathione (µM)	479·1	425·6, 532·7	398·1	345·6, 450·5	–81·1	–146·7, −15·4			0·016
WB GSH (µM)	446·1	386·3, 505·8	308·7	249·7, 367·6	–6·9	–10·6, −3·2			< 0·001
WB GSSG (µM)	26·9	19·5, 37·3	75·2	54·5, 103·5			2·8	1·9, 4·2	< 0·001
WB GSH:GSSG	16	10·5, 24·2	3·7	2·4, 5·6			0·2	0·1, 0·4	< 0·001
Plasma GLY (µM)	373·8	352·4, 395·1	377	356·3, 397·7	3·2	–22·9, 29·3			0·810
Plasma CYS (µM)	21	19·2, 23·1	20·8	19·1, 22·8			1	0·9, 1·1	0·876
Plasma methionine (µM)	52·4	46·6, 58·2	50·4	44·7, 56	–2·03	–9·13, 5·08			0·577

GSH, reduced glutathione; GSSG, oxidised glutathione; GSH:GSSG, reduced to oxidised glutathione ratio.

Values are means and 95 % CI of the mean (*P* ≤ 0·05).

#### Plasma glycine, cysteine and methionine profiles

No significant differences between age groups were observed in the plasma concentrations of free GLY (*P* = 0·81), CYS (*P* = 0·88) and methionine (*P* = 0·58) (Table 1). Free concentrations of all three AA were within reference ranges for plasma AA in healthy adult cats^(33)^.

### Comparing different levels of glycine supplementation (glycine inclusion level study)

Of the fifty-two senior cats enrolled for the dosing study, one cat was removed due to healthcare concerns unrelated to the study.

#### Plasma and erythrocyte glycine

For plasma, free GLY levels were significantly different between the final and baseline timepoints at levels of 1·5 % (difference 47·1, 95 % CI 24·4, 69·8 µM) and 6·0 % (difference 179, 95 % CI 156, 201 µM) free GLY dietary supplementation (all *P* < 0·001) (Table 2). A difference was not observed in free plasma GLY for the 0·5 % free GLY inclusion level (*P* = 0·9) (Table 2). Dietary-free GLY supplementation at 0·5 % (difference 94·2, 95 % CI 55·2, 133·0 µM), 1·5 % (difference 86, 95 % CI 46·9, 125·0 µM) and 6·0 % (difference 174, 95 % CI 136, 213·0 µM) led to significant differences in free erythrocyte GLY levels between final and baseline timepoints (all *P* < 0·001) (Table 2).

**Table 2.** Free plasma and erythrocyte glycine (GLY) levels from the effect of the GLY inclusion level study diet groups

% Dosing free GLY	GLY (µM)	Baseline		Final		Difference (final – baseline) in means		*P*
		Mean	95 % CI	Mean	95 % CI	Mean	95 % CI	
0 %	Plasma GLY	355	334, 376	355	334, 376	0·1	–22·4, 22·6	1
0 %	Erythrocyte GLY	282	251, 312	280	250, 311	–1·1	–39·8, 37·6	1
0·5 %	Plasma GLY	359	337, 380	380	358, 401	20·8	–1·8, 43·4	0·086
0·5 %	Erythrocyte GLY	253	222, 283	347	316, 378	94·2	55·2, 133	< 0·001
1·5 %	Plasma GLY	354	333, 376	401	380, 423	47·1	24·4, 69·8	< 0·001
1·5 %	Erythrocyte GLY	246	215, 277	332	301, 363	86	46·9, 125	< 0·001
6·0 %	Plasma GLY	365	343, 386	544	522, 565	179	156, 201	< 0·001
6·0 %	Erythrocyte GLY	268	237, 298	442	411, 473	174	136, 213	< 0·001

Values are means and 95 % CI of the mean (*P* ≤ 0·05).

These data informed the selection of the 1·5 % GLY as-fed dietary supplementation level for the subsequent feeding study.

### Impact of glycine supplementation on glutathione and markers of oxidative stress (glycine feeding study)

Of the forty-four senior cats recruited to the main feeding study, ten (seven female, three male) were removed during the 16-week trial, all due to poor consumption of the study diets, leading to weight loss that exceeded the threshold limits defined for the study (–10 % ideal BCS). This comprised the removal of two control cats (both female) during the acclimation phase, and three control cats (two female, one male) and five test cats (three female, two male) during the test phase, totalling five control and five test cats. This meant the feeding study completed with > 75 % power against the primary measure, erythrocyte total glutathione, to detect a 40 % difference between the groups. Data from cats removed during the feeding study were excluded from the statistical analysis of the study measures. Body weights of the thirty-four senior cats were not significantly different between the diet groups throughout the supplementation study (*P* = 0·62) (online Supplementary Fig. 1).

#### Erythrocyte glutathione

**Fig. 3.**
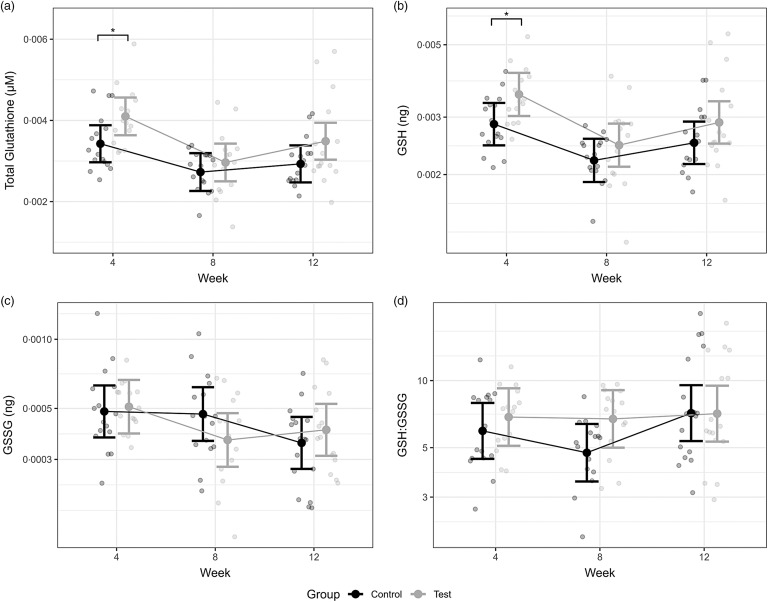
Mean erythrocyte glutathione concentrations for the GLY feeding study test (supplemented) and control (unsupplemented) senior cats. Erythrocyte glutathione represented as (a) total glutathione, (b) reduced glutathione (GSH), (c) oxidised glutathione (GSSG) and (d) GST:GSSG. Individual data are shown as open circles and means as solid circles with 95 % CI. * indicates significance between groups (*P* ≤ 0·05).

The dietary intervention influenced erythrocyte glutathione; total glutathione concentrations were found to be significantly higher in the GLY supplemented group than the control group at week 4 (difference 0·7, 95 % CI 0·1, 1·3 nM; *P* = 0·02) (Fig. 3(a), Table 3). Additionally, at week 4, significantly higher erythrocyte GSH levels were observed with 1·5 % free GLY supplementation (difference 1·2, 95 % CI 1, 1·5 ng; *P* = 0·03) (Fig. 3(b), Table 3). No further statistically significant differences were identified at the remaining test phase sampling points for erythrocyte total glutathione or GSH, although a trend not meeting the statistical level of significance was observed for total glutathione, with higher concentrations in the test diet group compared with the control diet group at week 12 (*P* = 0·06). Statistically significant differences were also not found for erythrocyte GSSH or GSH:GSSG between the control and free GLY supplemented groups (*P* ≥ 0·12) (Fig. 3(c) and (d), Table 3).

**Table 3.** Erythrocyte glutathione and glycine (GLY) concentrations, and free plasma GLY and cysteine (CYS) levels in the GLY feeding study test (supplemented) and control (unsupplemented) senior cats

Measure	Test phase week	Test		Control		Difference (test – control) in means		Fold change (test/control) in means		*P*
		Mean	95 % CI	Mean	95 % CI	Mean	95 % CI	Mean	95 % CI	
Erythrocyte total glutathione (µM)	4	0·0041	0·0037, 0·0046	0·0034	0·0030, 0·0039	0·0007	0·0001, 0·0013			0·020
Erythrocyte total glutathione (µM)	8	0·0030	0·0025, 0·0034	0·0027	0·0023, 0·0032	0·0002	–0·0004, 0·0008			0·659
Erythrocyte total glutathione (µM)	12	0·0035	0·0030, 0·0039	0·0029	0·0025, 0·0034	0·0006	0·0000, 0·0011			0·063
Erythrocyte GSH (ng)	4	0·0035	0·0030, 0·0041	0·0029	0·0025, 0·0033	1·23	1·0200, 1·5000			0·029
Erythrocyte GSH (ng)	8	0·0025	0·0021, 0·0029	0·0022	0·0019, 0·0026	1·11	0·9170, 1·3500			0·410
Erythrocyte GSH (ng)	12	0·0029	0·0025, 0·0034	0·0025	0·0025, 0·0029	1·15	0·954, 1·4000			0·182
Erythrocyte GSSG (ng)	4	0·0005	0·0004, 0·0007	0·0005	0·0004, 0·0006			1·0500	0·7500, 1·4700	0·982
Erythrocyte GSSG (ng)	8	0·0004	0·0003, 0·0005	0·0005	0·0004, 0·0006			0·7700	0·5500, 1·0900	0·201
Erythrocyte GSSG (ng)	12	0·0004	0·0003, 0·0005	0·0004	0·0003, 0·0005			1·1400	0·8160, 1·5900	0·720
GSH:GSSG	4	6·86	5·10, 9·24	5·95	4·45, 7·94			0·965	0·701, 1·33	0·987
GSH:GSSG	8	6·73	5·00, 9·06	4·75	3·52, 6·39			1·15	0·796, 1·67	0·689
GSH:GSSG	12	7·11	5·32, 9·49	7·15	5·35, 9·54			1·13	0·781, 1·64	0·770
Erythrocyte GLY (µM)	4	295	277, 312	246	229, 263	48·7	26·5, 70·8			< 0·001
Erythrocyte GLY (µM)	8	280	262, 297	259	241, 276	21·3	–0·9, 43·5			0·063
Erythrocyte GLY (µM)	12	283	266, 300	248	231, 265	35·2	13·3, 57·1			< 0·001
Plasma GLY (µM)	4	399	373, 424	345	319, 371	53·7	20·9, 86·6			< 0·001
Plasma GLY (µM)	8	407	380, 433	358	332, 384	48·6	15·4, 81·8			0·002
Plasma GLY (µM)	12	398	373, 423	343	317, 369	55·3	22·8, 87·8			< 0·001

GSH, reduced glutathione; GSSG, oxidised glutathione; GSH:GSSG, reduced to oxidised glutathione ratio.

All values are means and 95 % CI of the mean (*P* ≤ 0·05).

#### Leucocyte glutathione

The impact of 1·5 % free GLY supplementation on glutathione concentration within leucocytes was assessed in specific cell populations: CD4 + T-cells, CD8 + T-cells, CD14+ monocytes, CD21 + B-cells and granulocytes, via flow cytometry (online Supplementary Table 2). One statistically significant difference was identified; the absolute number of CD21+ cells (a marker for B-cells) was higher in the test group compared with the control group at week 8 of the test phase (difference 463, 95 % CI 188, 738·0 cells/µl; *P* < 0·001). No further statistically significant differences in glutathione concentration were observed, measured either via the number of cells of each subset per µl or the quantity of GSH per cell.

#### Erythrocyte and plasma amino acids

**Fig. 4.**
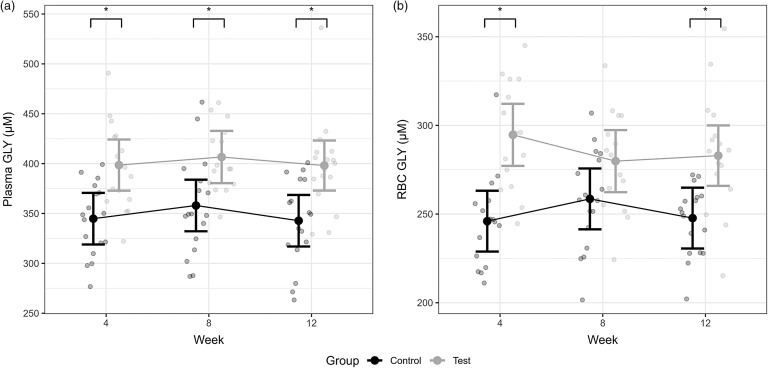
Mean (a) plasma and (b) erythrocyte glycine (GLY) levels from the effect of the GLY feeding study diet groups. Individual data are shown as open circles and means as solid circles with 95 % CI. * indicates significance between groups (*P* ≤ 0·05).

Levels of free GLY were found to differ between the test and control diet groups in the GLY supplementation study (Fig. 4) (Table 3). Free plasma GLY levels were significantly higher in the test group at all sampling timepoints in the test phase compared with the control group (*P* ≤ 0·009, Table 3) (Fig. 4(a), Table 3). Free erythrocyte GLY was found to be significantly higher for the test group at the first and last test phase timepoints compared with the control group (*P* ≤ 0·004, Table 3 (Fig. 4(b)) (Table 3). Aside from GLY, several AA profiled from plasma and erythrocytes indicated a statistically significant difference between diet groups only at one or two of the study’s sampling timepoints (online Supplementary Table 3). For plasma, these instances were observed at week 8 for free l-isoleucine, l-leucine, l-lysine, l-ornithine and l-valine, with levels of these amino acids being significantly higher in the test group compared with the control group (all *P*-values ≤ 0·024 and maximum difference of 19·2 µM) (online Supplementary Table 3a). Additionally, concentrations of free l-serine at week 4 (difference 1·1, 95 % CI 1, 1·3 µM; *P* = 0·04) and l-ornithine at week 12 (difference 1·2, 95 % CI 1, 1·4 µM; *P* = 0·02) were significantly higher in the test group compared with the control group within plasma (online Supplementary Table 3a). In contrast, levels of free l-asparagine, l-histidine, methionine and l-proline in erythrocytes were found to be lower at week 8 in the test compared with the control study group (all *P*-values ≤ 0·044 and maximum difference of −5·3 µM) (online Supplementary Table 3b).

#### Lymphoproliferative response

Cats offered the diet supplemented with 1·5 % free GLY had ConA-induced lymphoproliferative activities not significantly different to control group cats throughout the test phase (all *P* ≥ 0·35) (Table 4).

**Table 4.** Oxidative stress measure concentrations in the GLY feeding study test (supplemented) and control (unsupplemented) senior cats

Measure	Test Phase Week	Test		Control		Difference (test – control) in means		*P*
		Mean	95 % CI	Mean	95 % CI	Mean	95 % CI	
ConA (10 µg/ml)	4	0·95	0·80, 1·13	1·08	0·91, 1·28	0·88	0·70, 1·10	0·385
ConA (10 µg/ml)	8	0·87	0·73, 1	0·88	0·74, 1·05	0·99	0·79, 1·25	1·000
ConA (10 µg/ml)	12	0·97	0·80, 1·17	0·99	0·82, 1·18	0·99	0·78, 1·25	0·988
ConA (1 µg/ml)	4	1·12	0·97, 1·28	1·24	1·08, 1·42	0·908	0·75, 1·07	0·352
ConA (1 µg/ml)	8	1·08	0·94, 1·25	1·07	0·93, 1·23	1·01	0·84, 1·21	0·998
ConA (1 µg/ml)	12	1·24	1·07, 1·45	1·17	1·01, 1·36	1·06	0·88, 1·29	0·804
SAA	4	1·92	1·23, 3·02	3·1	1·98, 4·87	0·621	0·35, 1·11	0·128
SAA	8	1·65	1·04, 2·64	2·45	1·51, 3·97	0·68	0·37, 1·24	0·286
SAA	12	1·49	0·93, 2·37	2·3	1·45, 3·67	0·65	0·36, 1·17	0·198
8-OHdG (nG/ml)	4	30·3	24·8, 37	37·4	30·7, 45·6	0·81	0·63, 1·05	0·130
8-OHdG (nG/ml)	8	26·6	21·8, 32·5	37·6	30·8, 45·8	0·71	0·56, 0·91	0·004
8-OHdG (nG/ml)	12	31·4	25·8, 38·2	33·9	27·8, 41·3	0·93	0·72, 1·19	0·821
8-iso-PGF_2*α*_ (nG/mG Cr)	12	0·84	0·57, 1·10	1·16	0·88, 1·43	–0·32	–0·65, 0·01	0·058
PGF_2*α*_ (nG/mG Cr)	12	4·71	3·20, 6·94	7·66	5·14, 11·40	0·62	0·38, 1·00	0·049
5-series F2-IsoP (nG/mG Cr)	12	1·68	1·27, 2·21	2·09	1·58, 2·77	0·80	0·57, 1·13	0·197
2,3-dinor-5,6-dihydro-8isoPGF_2*α*_ (nG/mg Cr)	12	6·65	5·02, 8·80	9·63	7·21, 12·9	0·69	0·49, 0·98	0·040

ConA, concanavalin A; Cr, creatinine; SAA, serum amyloid A; 8-OHdG, 8-hydroxy-2’deoxyguanosine.

All values are means and 95 % CI of the mean (*P* ≤ 0·05).

#### Serum amyloid A

Concentrations of SAA were not significantly different between the diet groups throughout the supplementation study (all *P* ≥ 0·13) (Table 4).

#### Oxidative damage markers

Dietary supplementation of GLY was found to influence 8-OHdG and some of the urine F_2_-IsoPs measured as markers of oxidative damage (Table 4). The concentration of 8-OHdG was observed to be significantly lower for the test cats compared with the control group at week 8 (difference 0·7, 95 % CI 0·6, 0·9 nG/ml; *P* = 0·004) but was not found to be significantly different between the groups at the beginning and end of the dietary supplementation phase (*P* ≥ 0·13) (Table 4). Of the four F_2_-IsoPs quantified, comparisons between the study groups identified statistically significant differences for both PGF_2*α*_ (difference 0·6, 95 % CI 0·4, 1·0 ng/mg creatinine (Cr); *P* < 0·05) and 2,3-dinor-5,6-dihydro-8isoPGF_2*α*_ (difference 0·7, 95 % CI 0·5, 1·0 ng/mg Cr; *P* = 0·04), with levels lower for the GLY supplemented group compared with the control group (Table 4).

#### Biochemistry and haematology

A small number of parameters within the biochemistry panel were statistically significant between diet groups (online Supplementary Table 4). Cholesterol levels were significantly lower for the test group compared with the control group at weeks 4 and 8 (*P* ≤ 0·01). At week 8, symmetric dimethylarginine, Na:K ratio and creatine kinase were significantly higher (*P* ≤ 0·03), while phosphorous and cholesterol levels were significantly lower for the test group compared with the control group. At week 12, levels of symmetric dimethylarginine and blood urea nitrogen were found to be significantly higher in the test group compared with the control group (*P* ≤ 0·03).

Among the spectrum of parameters measured in the haematology analysis, only four significant differences were identified (online Supplementary Table 5). These were two basophil parameters at week 4, where both Basophils and % Basophils were significantly higher in the test group compared with the control group (*P* ≤ 0·004), and at week 12 where MCV was significantly higher and MCHC was significantly lower in the test group compared with the control group (*P* ≤ 0·04).

## Discussion

Glutathione is an antioxidant, important to the maintenance of oxidative defence, which, in turn, governs cellular survival. This series of investigations set out to establish whether glutathione is impacted by ageing in cats, and if supplementation with glutathione precursor GLY could alter circulating GLY and glutathione levels, and markers of oxidative stress over a 12-week dietary supplementation period in healthy, senior cats.

The preliminary, cross-sectional study indicated that senior cats have reduced WB and erythrocyte levels of glutathione compared with younger adult cats. Both forms of the antioxidant, GSH and GSSG, were determined. In the reduced state, GSH can react with CYS within proteins to maintain their reduced forms or with reactive oxygen species to neutralise them. The latter causes GSH to become reactive and form a disulphide bridge with another reactive GSH molecule to form a GSSH disulphide^(41)^. GSH can be regenerated from GSSG by glutathione reductase in the presence of NADPH. In healthy cells, approximately 90 % of the total glutathione pool is GSH, roughly 10 % GSSG and decreased GSH:GSSG is considered indicative of oxidative stress^(42)^. Significantly lower erythrocyte GSH:GSSG was observed for the senior cats compared with their younger counterparts, which may be indicative of greater oxidative stress. An imbalance in glutathione metabolism, defined by lower GSH:GSSG, has been linked to a number of chronic diseases including feline chronic renal failure^(43)^ and in humans, cancer, neurodegenerative disorders, cystic fibrosis, viral infection, diabetes mellitus, renal failure and liver disease^(44)^. Cats are thought to be particularly susceptible to oxidative injury because feline Hb contains 8–10 reactive sulfhydryl groups rather than 4 as in the dog and other mammalian species^(16,41,45,46)^. Therefore, cats may have a higher requirement for GSH to maintain sulfhydryl groups in the GSH form for the maintenance of normal function.

No significant age-related differences were observed in plasma levels of GLY, CYS or methionine. This may reflect the fact that all of the animals in the cross-sectional study were healthy and offered appropriate amounts of complete and balanced diets. As the plasma levels of GLY and CYS were within normal range for both age groups, the lower levels of glutathione in the senior cats may be due to age-related changes in the activity of enzymes involved in glutathione synthesis^(17)^. Reports in humans suggest that older individuals have lower intracellular levels of GLY and CYS due to slower body protein turnover or decreased *de novo* synthesis^(21,47)^. However, protein turnover in cats is suggested to be higher than in humans due to the reliance on protein metabolism for energy^(48)^. There is evidence for a deceleration of overall protein turnover with ageing in humans^(49)^, which suggests that the supply of GLY and CYS could be decreased. Impaired protein digestibility has been reported with age in cats^(50,51)^ which could affect intracellular amino acid levels and hence glutathione metabolism. In future studies, measuring erythrocyte levels of these amino acids could be more informative than plasma levels, particularly if investigating changes due to supplementation.

For the GLY feeding study, 1·5 % free GLY was selected for supplementation from the inclusion level study. Using the average body weight of a cat and average consumption, 1·75 mM/kg/d total GLY was fed in the GLY feeding study. This GLY level is roughly equivalent to the 1·33 mM/kg/d dose used in human dietary intervention studies^(21,23)^. In the GLY feeding study, levels of erythrocyte total glutathione and GSH were significantly elevated in the test group of senior cats receiving 1·5 % free GLY supplementation compared with the control group at week 4. No further statistically significant observations were identified in the feeding study. However, erythrocyte total glutathione indicated a trend towards higher concentration in the test group compared with the control group at week 12. Collectively, these findings suggest that supplementation with free GLY has some capacity to increase intracellular glutathione concentrations in healthy senior cats. Early in the supplementation phase, an excess of GLY may drive the reaction with χ-glutamylcysteine (χ–GC), increasing *de novo* generation of glutathione in the second part of a two-step reaction catalysed by GSH synthase^(52)^. The drive towards glutathione biosynthesis may continue until either one of two circumstances arise. Substrate availability of χ–GC declines, impacting the rate of the secondary enzymatic reaction via GSH synthase, further reducing glutathione synthesis. Alternatively, the glutathione pool could increase until a threshold has been reached, which elicits a competitive feedback inhibition, in which glutathione competes with glutamate for glutamate cysteine ligase, limiting the rate of the primary synthesis reaction^(53)^. Either consequence restricts further glutathione synthesis and could explain the subsequent decline in erythrocyte intracellular levels at week 8, where the study groups were found to have comparable glutathione levels. The trend observed at week 12 where higher erythrocyte total glutathione was observed in the test group might suggest the rate limiting condition being overcome, enabling the loop to cycle back to re-establish glutathione synthesis and increase intracellular concentrations once again.

Levels of glutathione observed in the GLY feeding study were not found to be comparable to the cross-sectional study. None of the individuals within the study cohorts was the same between the studies. Glutathione concentrations in the cross-sectional study were not normalised to Hb based on the assumption that variation in erythrocyte Hb levels in healthy cats, including seniors, is small^(54,55)^. This could provide an explanation for the sizeable difference observed. Another important consideration is the difference in methods for the measurement of glutathione. The MS-based approach (UPLC-MRM MS) used in the feeding study and the ELISA method utilised in the cross-sectional study are not comparable. Furthermore, it is acknowledged that the measurement of glutathione may be subject to inaccuracies due to the development of post-sampling artifacts^(56)^. Oxidation biases the pool of GSSG, leading to its overestimation^(56)^. Concentrations reported elsewhere for clinically normal client-owned cats with mean age 10·62 (sd 0·76) years are 4·24 (sd 0·67) mM for WB GSH and 19·66 (sd 2·75) µM for WB GSSH^(26)^.

The feeding study included the measurement of glutathione in leucocytes and markers of immune function, such as lymphocyte proliferation. There is evidence in humans that dietary supplementation with pressurised whey protein isolate can alter lymphocyte glutathione^(57)^. Glutathione has also been shown to play an important role in the activation of T-lymphocytes^(58)^. In addition, depletion of intracellular glutathione reduced lymphocyte differentiation and proliferation in humans and rodents^(59,60)^. Of particular relevance is the age-associated reduction in lymphoproliferative response to the T-lymphocyte specific polyclonal lymphocyte activator, ConA, which has been shown to be partially restored by dietary glutathione supplementation in mice^(61)^. However, here attempts to increase leucocyte glutathione (GSH) via dietary GLY supplementation were largely unsuccessful, although one statistically significant observation was noted for CD21+ cells at week 8. This suggests elevations in glutathione brought about by free GLY supplementation may not be limited to erythrocytes.

Along with other AA, levels of GLY were measured in both plasma and erythrocytes. This confirmed that the supplementation level selected was sufficient to significantly elevate plasma and erythrocyte GLY concentrations in the test group of senior cats throughout the 12-week test phase. Erythrocyte GLY levels were found to significantly differ between the study groups post-supplementation at weeks 4 and 12. However, statistical significance was not achieved at the intermediate sampling timepoint. These findings align with the observations for erythrocyte total glutathione and the associated hypothesis proposed. Higher erythrocyte GLY levels were observed at the same time as the elevations in intracellular glutathione concentrations at week 4 (*P* = 0·02) and week 12 (*P* = 0·063) of the supplementation phase. In between (week 8), concentrations of erythrocyte GLY were also higher, but not significantly different from the control group of senior cats and erythrocyte total glutathione was not statistically significant. Taken together, the rate of synthesis of the antioxidant may be declining. A mechanism to moderate AA uptake into erythrocytes could also be at play. Adaptive regulation for transport of neutral AA, including GLY, across membranes, has been reported for mammalian cells^(62)^. Another plausible explanation for the difference in the observations in erythrocyte GLY between the test phase timepoints is fluctuation in the intake of the test diet, with a possible decline after week 4, which is subsequently restored prior to week 12. However, diet consumption data collected for the feeding study confirmed the relative consistency in the intake of both diets (online Supplementary Fig. 2). Dietary supplementation of GLY significantly impacted levels of other AA in plasma and erythrocytes, predominantly at the intermediate timepoint measure. Similarly, enrichment of numerous AA including leucine, isoleucine, valine, ornithine and proline was determined in plasma in a study of GLY metabolism conducted via oral administration of ^15^N-GLY in young adult men^(63)^. Given the hypothesis proposed regarding reduced glutathione biosynthesis around this timeframe within our study, conversion of excess GLY to other amino acids offers a plausible explanation.

To explore the impact of dietary GLY supplementation beyond glutathione, several markers of oxidative stress, shown to be clinically relevant in ageing^(64)^ were explored. Acute phase proteins have been shown to positively correlate with erythrocyte glutathione concentration in humans^(65)^. One such acute phase protein, SAA, has been reported to be a more responsive marker of inflammation than C-reactive protein in cats^(66)^. Levels of SAA could not be differentiated between the feeding study groups. Other markers, namely F_2_-IsoPs, have been publicised as the best available indicators of oxidative stress for humans. Furthermore, F_2_-IsoPs have been shown to increase in different biofluids over the human lifespan^(67)^ and to be higher in elderly versus younger subjects^(21)^. Measurement of F_2_-IsoPs is commonly performed in urine due to their chemical stability brought about by a lack of artificial auto-oxidation^(68)^. Urine PGF_2*α*_ and 2,3-dinor-5,6-dihydro-8iso PGF_2*α*_ were significantly lower in the test group compared with the control group, following 12 weeks of 1·5 % free GLY supplementation. Produced during periods of inflammation and oxidative stress, prostaglandin F_2*α*_ (PGF_2*α*_) is an eicosanoid, which promotes the formation of 8-iso-PGF_2*α*_ and 2,3-dinor-5,6-dihydro-8iso PGF_2*α*_^(69,70)^. In addition, although not meeting the level of statistical significance (*P* = 0·06), there was a trend towards lower urinary 8-iso-PGF_2*α*_ in the test group. Our findings indicate a reduction in these markers of oxidative stress in senior cats eating a diet supplemented with free GLY. This is consistent with the human intervention study by Sekhar *et al.* in which dietary GLY and CYS supplementation, targeting age-associated glutathione decline, significantly lowered F_2_-IsoPs^(21)^. Further oxidative damage, to DNA specifically, was investigated via plasma 8-OHdG, one of the best measures of the mutagenic consequences of oxidative stress^(31)^. Human studies provide evidence that 8-OHdG levels increase with age^(28,71)^. A significant decrease in 8-OHdG was identified at week 8 in the test group compared with the control group, which was not apparent at week 4 or 12. As with lymphocyte glutathione, these findings align with intervention studies conducted in other species; dietary supplementation with glutathione^(72)^ and vitamins E and C^(73)^ have been shown to reduce 8-OHdG in diabetic patients^(72)^ and aged rats^(73)^, respectively. Taken together, these results suggest dietary supplementation of GLY alters markers of oxidative stress in senior cats, which appears to be dependent on an increase in erythrocyte glutathione. These findings align with a review by Zhong *et al.* ^(74)^, who discuss the protective effects of GLY, suggesting it as a novel anti-inflammatory, immunomodulatory and cytoprotective agent. For oxidative stress, they propose various mechanisms by which GLY prevents reactive oxygen species formation^(74)^.

The results of the feeding study suggest dietary supplementation with 1·5 % free GLY, a precursor of glutathione, may offer a viable route to alleviating the age-associated reduction in glutathione observed in senior cats. Further work is needed to elucidate the mechanism(s) by which this occurs. As discussed earlier, *de novo* glutathione synthesis is dependent on sufficient availability of GLY and χ–GC. In turn, the pool of χ–GC is dependent on a prior reaction with glutamate cysteine ligase, requiring glutamate and CYS^(51)^. Supplementation with χ–GC in human and rodent studies results in increased glutathione, confirming biosynthesis of glutathione can occur without the first enzyme reaction^(75–77)^. Therefore, CYS could be another limiting factor in the synthesis of glutathione, although supplementation with CYS must be carefully considered due to its possible toxicity^(78)^. Unfortunately, this could not be assessed as for technical reasons, levels of CYS were not determined in the feeding study. Exploring mechanisms by which GLY may reduce oxidative stress in senior cats in conjunction with other glutathione precursors is therefore warranted.

Other than the limitations discussed thus far, increased numbers of animals completing the study would have improved the ability to detect a difference in the supplementation study. Notably, two trends were observed within the study, one for erythrocyte total glutathione (*P* = 0·06) and another for one of the F_2_-IsoPs (*P* = 0·06), both week 12 (end) measures. Such trends might have led to statistically significant differences if a larger senior feline cohort had been used.

In summary, we have been able to confirm that levels of glutathione, the most abundant antioxidant, are significantly lower in WB and erythrocytes in older, senior cats compared with younger adult cats. Offering older cats a dry diet supplemented with 1·5 % as-fed free GLY for 12 weeks induced significant elevations in erythrocyte total glutathione and GSH, although this was limited to early in the supplementation phase. Supplementation of GLY was also found to influence leucocyte GSH levels and markers of oxidative stress, although only at certain timepoints within the study. Overall, the results suggest age-associated reduction in erythrocyte glutathione in cats may be partially resolved by dietary free GLY supplementation at the concentration tested. Further studies investigating the mechanism(s) of action of GLY and the impact of supplementation with other glutathione precursors are needed.

## Supporting information

Ruparell et al. supplementary material 1 Ruparell et al. supplementary material

Ruparell et al. supplementary material 2 Ruparell et al. supplementary material

Ruparell et al. supplementary material 3 Ruparell et al. supplementary material

Ruparell et al. supplementary material 4 Ruparell et al. supplementary material

Ruparell et al. supplementary material 5 Ruparell et al. supplementary material

Ruparell et al. supplementary material 6 Ruparell et al. supplementary material

Ruparell et al. supplementary material 7 Ruparell et al. supplementary material

## Supplementary material

For supplementary materials referred to in this article, please visit https://doi.org/10.1017/S0007114524000370
