# Supplementary figures and images for: Glycine supplementation can partially restore oxidative stress-associated glutathione deficiency in ageing cats
Source: Br J Nutr. 2024 Feb 29;131(12):1947–61. doi: 10.1017/S0007114524000370 (PMC11361917; doi:10.1017/S0007114524000370)

**Supplementary Figure 1. Average weekly bodyweight of GLY feeding study cats by diet**
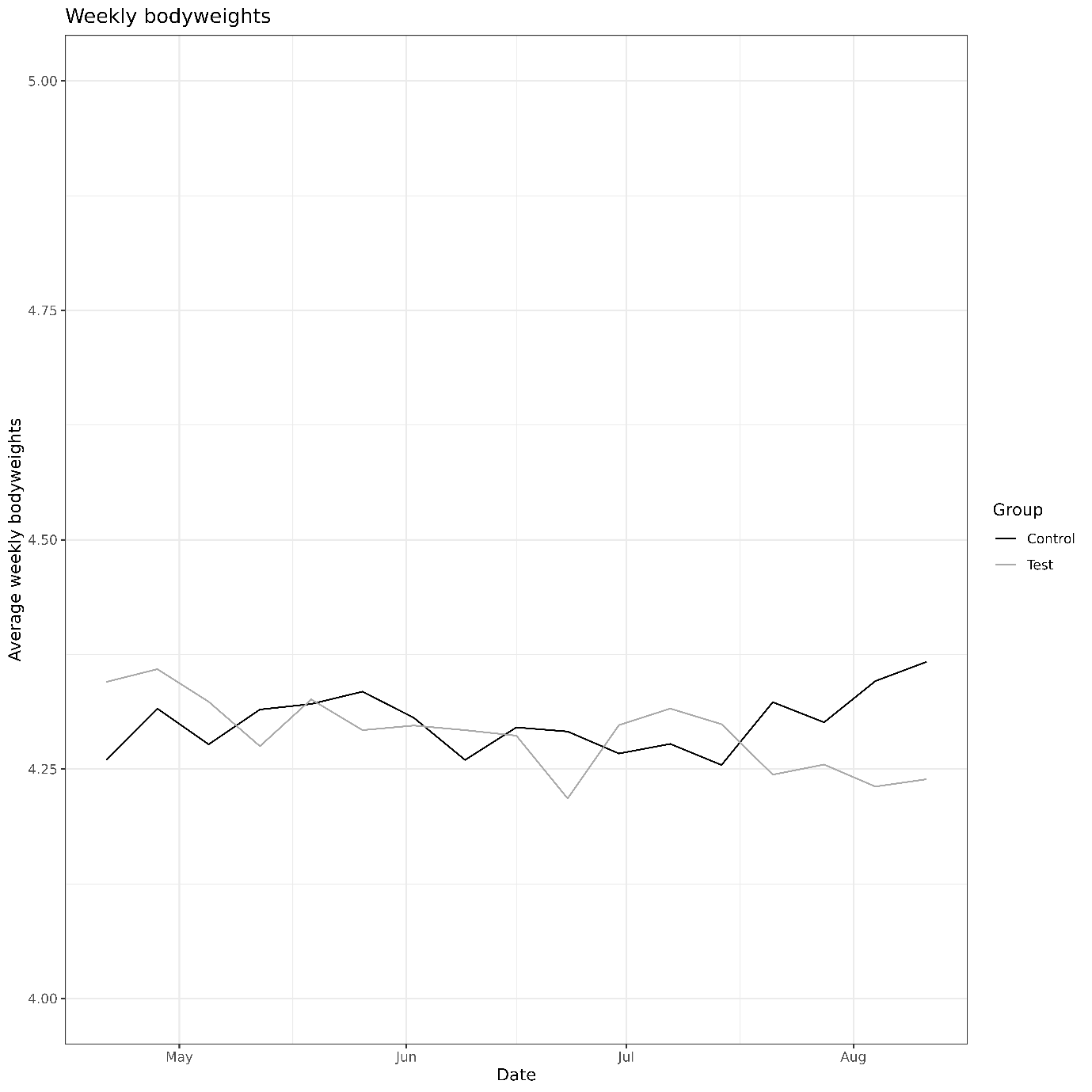

Supplement: Ruparell et al. supplementary material 1 — Ruparell et al. supplementary material [file S0007114524000370sup001.docx]

**Supplementary Figure 2. Average (7-day rolling) daily intake of GLY feeding study diets.**


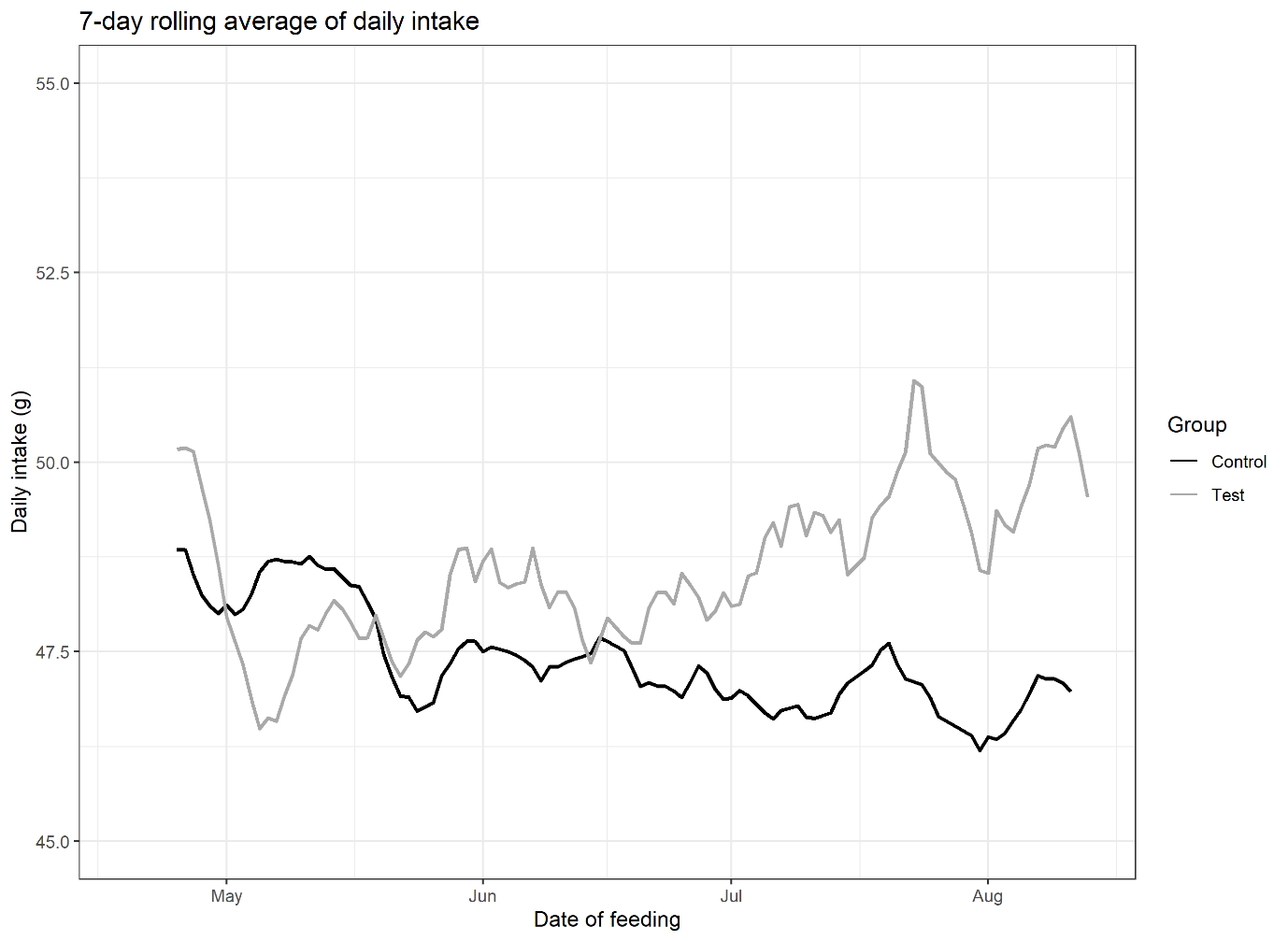

Supplement: Ruparell et al. supplementary material 2 — Ruparell et al. supplementary material [file S0007114524000370sup002.docx]
