# Supplementary material for: Glycine supplementation can partially restore oxidative stress-associated glutathione deficiency in ageing cats
Source: Br J Nutr. 2024 Feb 29;131(12):1947–61. doi: 10.1017/S0007114524000370 (PMC11361917; doi:10.1017/S0007114524000370)
Supplement: Ruparell et al. supplementary material 3 — Ruparell et al. supplementary material [file S0007114524000370sup003.docx]

**Supplementary Table 1. Nutrient composition of the glycine (GLY) inclusion level and feeding study diets.**

|  |  | GLY inclusion study | | | | GLY feeding study | |
| --- | --- | --- | --- | --- | --- | --- | --- |
| Nutrient | Unit | 0% free GLY (Control diet) | 0.5% free GLY | 1.5% free GLY | 6% free GLY | 0% free GLY (Control diet) | 1.5% free GLY |
| Energy | kcal/g | 3681 | 3653 | 3653 | 3679 | 3792 | 3815 |
| Moisture | % as-fed | 7.06 | 7.70 | 7.61 | 7.56 | 6.39 | 6.19 |
| Protein | % as-fed | 34.56 | 34.75 | 35.56 | 40.44 | 34.31 | 35.69 |
| Fat | % as-fed | 13.79 | 13.60 | 13.53 | 14.03 | 15.71 | 15.76 |
| Crude Fiber | % as-fed | 1.5 | 1.3 | 1.4 | 1.3 | 1.2 | 1.2 |
| Ash | % as-fed | 5.97 | 6.05 | 5.96 | 6.06 | 6.52 | 6.13 |
| Total GLY | % as-fed | 2.36 | 2.89 | 3.96 | 8.39 | 2.42 | 3.94 |
| Free GLY | % as-fed | 0.03 | 0.54 | 1.57 | 6.02 | 0.03 | 1.54 |
| Cystine* | % as-fed | 0.42 | 0.43 | 0.43 | 0.42 | 0.41 | 0.42 |
| Methionine | % as-fed | 0.89 | 0.88 | 0.88 | 0.88 | 0.91 | 0.89 |

GLY, glycine.
* a combination of cystine and cysteine.
Amino acids are total, unless otherwise stated.
