# Supplementary material for: Glycine supplementation can partially restore oxidative stress-associated glutathione deficiency in ageing cats
Source: Br J Nutr. 2024 Feb 29;131(12):1947–61. doi: 10.1017/S0007114524000370 (PMC11361917; doi:10.1017/S0007114524000370)
Supplement: Ruparell et al. supplementary material 4 — Ruparell et al. supplementary material [file S0007114524000370sup004.doc]

**Supplementary Table 2. White blood cell glutathione concentrations in the GLY feeding study test (supplemented) and control (unsupplemented) senior cats.**

**(a) Absolute cell counts (cells/µL)**

| WBC cell population | Test Phase Week | Test | Control | Difference (Test – Control) in Means | Fold Change (Test / Control) in Means | *P* value |
| --- | --- | --- | --- | --- | --- | --- |
| CD4+ | 8 | 748 (590, 906) | 677 (524, 831) | 70.9 (-126.0, 268.0) |  | 0.616 |
| CD4+ | 12 | 825 (673, 978) | 789 (633, 945) | 36.2 (-159.0, 231.0) |  | 0.872 |
| CD8+ | 8 | 254 (192, 336) | 277 (211, 363) |  | 0.9 (0.7, 1.3) | 0.813 |
| CD8+ | 12 | 312 (239, 407) | 328 (248, 432) |  | 1.0 (0.7, 1.3) | 0.962 |
| CD14+ | 8 | 634 (384, 1046) | 549 (338, 891) |  | 1.2 (0.6, 2.2) | 0.846 |
| CD14+ | 12 | 602 (376, 962) | 618 (374, 1021) |  | 0.97 (0.5, 1.8) | 0.994 |
| CD21+ | 8 | 1128 (907, 1349) | 666 (452, 879) | 463 (188, 738) |  | <0.001 |
| CD21+ | 12 | 1054 (843, 1265) | 908 (689, 1127) | 146 (-126, 418) |  | 0.384 |
| Granulocytes | 8 | 8387 (6351, 10444) | 8266 (6287, 10246) | 131 (-2424, 2686) |  | 0.991 |
| Granulocytes | 12 | 7775 (5843, 9706) | 8336 (6296, 10376) | -561 (-3081, 1959) |  | 0.849 |

**(b) Mean quantification per cell (g x 10^-13^)**

| WBC cell population | Test Phase Week | Test | Control | Difference (Test – Control) in Means | Fold Change (Test / Control) in Means | *P* value |
| --- | --- | --- | --- | --- | --- | --- |
| CD4+ | 8 | 3.02 (3.00, 3.04) | 3.02 (2.99, 3.04) |  | 1 (0.99, 1.01) | 0.889 |
| CD4+ | 12 | 3.01 (2.99, 3.03) | 3.00 (2.98, 3.03) |  | 1 (0.99, 1.01) | 0.946 |
| CD8+ | 8 | 2.99 (2.97, 3.01) | 2.99 (2.97, 3.01) |  | 1 (0.99, 1.01) | 0.997 |
| CD8+ | 12 | 2.97 (2.95, 2.99) | 2.98 (2.96, 3.00) |  | 1.00 (0.99, 1.01) | 0.697 |
| CD14+ | 8 | 6.53 (6.17, 6.89) | 6.72 (6.37, 7.07) | -0.19 (-0.64, 0.26) |  | 0.538 |
| CD14+ | 12 | 6.42 (6.07, 6.76) | 6.45 (6.10, 6.81) | -0.04 (-0.48, 0.40) |  | 0.973 |
| CD21+ | 8 | 2.94 (2.92, 2.96) | 2.96 (2.94, 2.97) | -0.02 (-0.04, 0.00) |  | 0.084 |
| CD21+ | 12 | 2.93 (2.91, 2.94) | 2.94 (2.93, 2.96) | -0.02 (-0.04, 0.00) |  | 0.108 |
| Granulocytes | 8 | 5.62 (5.32, 5.93) | 5.87 (5.58, 6.17) | -0.25 (-0.63, 0.13) |  | 0.262 |
| Granulocytes | 12 | 5.36 (5.07, 5.64) | 5.34 (5.04, 5.65) | 0.01 (-0.36, 0.39) |  | 0.996 |

All values are means and brackets indicate 95% confidence intervals of the mean (*P* ≤ 0.05). Brackets indicate 95% confidence intervals of the mean.
