# Supplementary material for: Glycine supplementation can partially restore oxidative stress-associated glutathione deficiency in ageing cats
Source: Br J Nutr. 2024 Feb 29;131(12):1947–61. doi: 10.1017/S0007114524000370 (PMC11361917; doi:10.1017/S0007114524000370)
Supplement: Ruparell et al. supplementary material 5 — Ruparell et al. supplementary material [file S0007114524000370sup005.doc]

**Supplementary Table 3. Free plasma and red blood cell amino acid concentrations in the GLY feeding study test (supplemented) and control (unsupplemented) senior cats.
(a) Plasma**

| Parameter (μM) | Test Phase Week | Test | Control | Difference (Test – Control) in Means | Fold Change (Test / Control) in Means | *P* value |
| --- | --- | --- | --- | --- | --- | --- |
| 1–Methyl–L–Histidine *1–mhis* | 4 | 16.8 (15.5, 18.1) | 18.3 (16.9, 19.7) |  | 0.9 (0.8, 1.0) | 0.121 |
| 1–Methyl–L–Histidine *1–mhis* | 8 | 16.5 (15.3, 17.9) | 18.3 (16.9, 19.8) |  | 0.9 (0.8, 1.0) | 0.046 |
| 1–Methyl–L–Histidine *1–mhis* | 12 | 14.6 (13.5, 15.7) | 15.5 (14.3, 16.7) |  | 0.9 (0.9, 1.0) | 0.387 |
| 3–Methyl–L–Histidine *3–mhis* | 4 | 8.7 (7.1, 10.6) | 8.8 (7.2, 10.8) |  | 1.0 (0.8, 1.3) | 1.000 |
| 3–Methyl–L–Histidine *3–mhis* | 8 | 8.0 (6.52, 9.81) | 8.1 (6.6, 9.9) |  | 1.0 (0.8, 1.3) | 0.999 |
| 3–Methyl–L–Histidine *3–mhis* | 12 | 7.6 (6.3, 9.2) | 7.1 (5.8, 8.6) |  | 1.1 (0.8, 1.4) | 0.848 |
| L–Alanine *Ala* | 4 | 466 (411.0, 522.0) | 483 (427.0, 539.0) | -16.3 (-90.8, 58.2) |  | 0.921 |
| L–Alanine *Ala* | 8 | 556 (499.0, 613.0) | 504 (448.0, 560.0) | 51.4 (-23.9, 127.0) |  | 0.257 |
| L–Alanine *Ala* | 12 | 533 (479.0, 588.0) | 520 (464.0, 576.0) | 13.9 (-59.9, 87.6) |  | 0.948 |
| L–α–Amino–η Butyric acid *Aaba* | 4 | 9.9 (8.3, 11.4) | 9.4 (7.9, 10.9) | 0.5 (-1.5, 2.4) |  | 0.925 |
| L–α–Amino–η Butyric acid *Aaba* | 8 | 8.3 (6.69, 9.84) | 7.29 (5.8, 8.8) | 1.0 (-1.1, 3.0) |  | 0.568 |
| L–α–Amino–η Butyric acid *Aaba* | 12 | 10.2 (8.76, 11.7) | 10.7 (9.1, 12.2) | -0.4 (-2.4, 1.6) |  | 0.938 |
| L–Arginine *Arg* | 4 | 84 (74.8, 93.2) | 90.6 (81.4, 99.9) | -6.7 (-18.4, 5.2) |  | 0.423 |
| L–Arginine *Arg* | 8 | 104 (95.0, 114.0) | 92.9 (83.6, 102.0) | 11.6 (-0.4, 23.5) |  | 0.061 |
| L–Arginine *Arg* | 12 | 98.7 (89.7, 108.0) | 94.2 (85, 103.0) | 4.5 (-7.2, 16.2) |  | 0.713 |
| L–Asparagine *Asn* | 4 | 88.3 (80.2, 96.3) | 93.5 (85.3, 102.0) | -5.2 (-15.5, 5.1) |  | 0.484 |
| L–Asparagine *Asn* | 8 | 101 (92.8, 109.0) | 99.1 (87.3, 103.0) | 1.9 (-8.5, 12.3) |  | 0.944 |
| L–Asparagine *Asn* | 12 | 95.7 (87.9, 104.0) | 95.4 (87.3, 103.0) | 0.4 (-9.8, 10.6) |  | 1.000 |
| L–Aspartic Acid *Asp* | 4 | 16.3 (14.8, 17.9) | 16.9 (15.3, 18.9) |  | 1.0 (0.9, 1.1) | 0.832 |
| L–Aspartic Acid *Asp* | 8 | 16.8 (15.2, 18.5) | 18 (16.3, 19.8) |  | 0.9 (0.8, 1.1) | 0.418 |
| L–Aspartic Acid *Asp* | 12 | 18.1 (16.4, 19.9) | 17.7 (16.1, 19.6) |  | 1.0 (0.9, 1.2) | 0.971 |
| L–Citrulline *Citr* | 4 | 15.4 (13.5, 17.5) | 16.3 (14.3, 18.6) |  | 0.9 (0.8, 1.1) | 0.747 |
| L–Citrulline *Citr* | 8 | 17.3 (15.1, 19.7) | 19.8 (17.4, 22.6) |  | 0.9 (0.7, 1.0) | 0.144 |
| L–Citrulline *Citr* | 12 | 16.3 (14.3, 18.5) | 15.1 (13.2, 17.2) |  | 1.1 (0.9, 1.3) | 0.578 |
| Cystine *Cys* | 4 | 15.7 (13.7, 17.6) | 13.9 (11.9, 15.8) | 1.8 (-0.7, 4.2) |  | 0.208 |
| Cystine *Cys* | 8 | 17.1 (15.1, 19.0) | 16.1 (14.2, 18.0) | 1.0 (-1.5, 3.5) |  | 0.655 |
| Cystine *Cys* | 12 | 16.6 (14.8, 18.5) | 15 (13.1, 16.9) | 1.7 (-0.8, 4.1) |  | 0.254 |
| Cystathione *Cysth* | 4 | 15.1 (13.3, 16.8) | 14.7 (12.9, 16.5) | 0.3 (-2.0, 2.6) |  | 0.975 |
| Cystathione *Cysth* | 8 | 14.8 (13, 16.5) | 15.8 (14, 17.6) | -1.1 (-3.3, 1.3) |  | 0.576 |
| Cystathione *Cysth* | 12 | 14.1 (12.4, 15.8) | 12.3 (10.5, 14.1) | 1.8 (-0.5, 4.1) |  | 0.157 |
| Glutamine *Gln* | 4 | 689.0 (647.0, 731.0) | 703.0 (660.0, 745.0) | -13.9 (-67.9, 40.2) |  | 0.876 |
| Glutamine *Gln* | 8 | 680.0 (637.0, 723) | 699 (656.0, 741.0) | -18.9 (-73.5, 35.6) |  | 0.749 |
| Glutamine *Gln* | 12 | 730.0 (689.0, 772.0) | 744 (702.0, 786.0) | -13.6 (-66.9, 39.8) |  | 0.879 |
| L–Glutamic acid *Glu* | 4 | 43.5 (38.5, 48.5) | 44.8 (39.7, 49.8) | -1.3 (-7.7, 5.1) |  | 0.947 |
| L–Glutamic acid *Glu* | 8 | 43.8 (38.7, 48.9) | 46.8 (41.8, 51.8) | -3.0 (-9.5, 3.5) |  | 0.583 |
| L–Glutamic acid *Glu* | 12 | 36.5 (31.7, 41.4) | 40.2 (35.2, 45.2) | -3.7 (-10.0, 2.6) |  | 0.396 |
| Glycine *Gly* | 4 | 399.0 (373.0, 424.0) | 345 (319.0, 371.0) | 53.7 (20.9, 86.6) |  | <0.001 |
| Glycine *Gly* | 8 | 407.0 (380.0, 433.0) | 358.0 (332.0, 384.0) | 48.6 (15.4, 81.8) |  | 0.002 |
| Glycine *Gly* | 12 | 398.0 (373.0, 423.0) | 343.0 (317.0, 369.0) | 55.3 (22.8, 87.8) |  | <0.001 |
| L–Histidine *His* | 4 | 122.0 (116.0, 128.0) | 128.0 (122.0, 135.0) |  | 0.9 (0.9, 1.0) | 0.120 |
| L–Histidine *His* | 8 | 130.0 (124.0, 137.0) | 134.0 (128.0, 141.0) |  | 1.0 (0.9, 1.0) | 0.624 |
| L–Histidine *His* | 12 | 132.0 (126.0, 138.0) | 130.0 (124.0, 137.0) |  | 1.0 (0.9, 1.1) | 0.966 |
| L–Isoleucine *Ile* | 4 | 57.3 (49.1, 65.6) | 58.4 (50.1, 66.7) | -1.0 (-11.7, 9.7) |  | 0.994 |
| L–Isoleucine *Ile* | 8 | 75.0 (66.5, 83.5) | 59.1 (50.8, 67.4) | 15.8 (5.0, 26.7) |  | 0.001 |
| L–Isoleucine *Ile* | 12 | 74.8 (66.8, 82.9) | 67.5 (59.2, 75.8) | 7.4 (-3.2, 17.9) |  | 0.258 |
| L–Leucine *Leu* | 4 | 114.0 (100.0, 127.0) | 115.0 (101.0, 128.0) | -0.9 (-17.9, 16.2) |  | 0.999 |
| L–Leucine *Leu* | 8 | 134.0 (120.0, 147.0) | 114.0 (101.0, 128.0) | 19.2 (1.9, 36.5) |  | 0.024 |
| L–Leucine *Leu* | 12 | 139.0 (126.0, 151.0) | 127.0 (114.0, 140.0) | 11.6 (-5.2, 28.4) |  | 0.269 |
| L–Lysine *Lys* | 4 | 123.0 (107.0, 139.0) | 130.0 (114.0, 146.0) | -7.1 (-27.8, 13.7) |  | 0.794 |
| L–Lysine *Lys* | 8 | 160.0 (144.0, 177.0) | 126.0 (109.0, 142.0) | 34.9 (13.8, 56.0) |  | <0.001 |
| L–Lysine *Lys* | 12 | 145.0 (129.0, 160.0) | 127.0 (111.0, 143.0) | 17.7 (-2.9, 38.4) |  | 0.114 |
| Methionine *Met* | 4 | 36.1 (32.5, 39.7) | 37 (33.4, 40.7) | -0.9 (-5.5, 3.8) |  | 0.947 |
| Methionine *Met* | 8 | 37.3 (33.6, 41.0) | 38.8 (35.2, 42.5) | -1.5 (-6.2, 3.2) |  | 0.797 |
| Methionine *Met* | 12 | 40.2 (36.6, 43.7) | 40.1 (36.5, 43.7) | 0.1 (-4.5, 4.7) |  | 1.000 |
| L–Ornithine *Orn* | 4 | 8.7 (7.6, 9.9) | 8.8 (7.7, 10.0) |  | 1.0 (0.8, 1.2) | 0.997 |
| L–Ornithine *Orn* | 8 | 10.5 (9.2, 12) | 8.1 (7.1, 9.3) |  | 1.3 (1.1, 1.5) | 0.001 |
| L–Ornithine *Orn* | 12 | 9.9 (8.7, 11.3) | 8.2 (7.2, 9.37) |  | 1.2 (1.0, 1.4) | 0.022 |
| L–Phenylalanine *Phe* | 4 | 66.5 (60.6, 73.1) | 67.8 (61.7, 74.4) |  | 1.0 (0.9, 1.1) | 0.976 |
| L–Phenylalanine *Phe* | 8 | 78.1 (70.9, 86) | 70.2 (63.9, 77.1) |  | 1.1 (1.0, 1.3) | 0.104 |
| L–Phenylalanine *Phe* | 12 | 76.9 (70.3, 84.2) | 70.9 (64.6, 77.8) |  | 1.1 (1.0, 1.2) | 0.267 |
| L–Proline *Pro* | 4 | 137.0 (123.0, 153.0) | 140.0 (126.0, 157.0) |  | 1.0 (0.8, 1.1) | 0.963 |
| L–Proline *Pro* | 8 | 136.0 (121.0, 152.0) | 143.0 (128.0, 160.0) |  | 1.0 (0.8, 1.1) | 0.689 |
| L–Proline *Pro* | 12 | 130.0 (117.0, 145.0) | 131.0 (118.0, 147.0) |  | 1.0 (0.9, 1.1) | 0.998 |
| L–Serine *Ser* | 4 | 185.0 (168.0, 204.0) | 162.0 (147.0, 179.0) |  | 1.1 (1.0, 1.3) | 0.038 |
| L–Serine *Ser* | 8 | 198.0 (179.0, 219.0) | 180.0 (163.0, 199.0) |  | 1.1 (1.0, 1.2) | 0.188 |
| L–Serine *Ser* | 12 | 178.0 (162.0, 196.0) | 162.0 (146.0, 179.0) |  | 1.1 (1.0, 1.3) | 0.168 |
| Taurine *Taur* | 4 | 131.0 (112.0, 152.0) | 138.0 (118.0, 160.0) |  | 0.9 (0.8, 1.2) | 0.885 |
| Taurine *Taur* | 8 | 142.0 (122.0, 167.0) | 140.0 (121.0, 164.0) |  | 1.0 (0.8, 1.2) | 0.998 |
| Taurine *Taur* | 12 | 125.0 (108.0, 145.0) | 144.0 (124.0, 168.0) |  | 0.9 (0.7, 1.1) | 0.212 |
| L–Threonine *Thr* | 4 | 99.6 (90.1, 109.0) | 103.0 (93.4, 112.0) | -3.3 (-15.5, 8.9) |  | 0.878 |
| L–Threonine *Thr* | 8 | 116.0 (106.0, 126.0) | 107.0 (97.3, 116.0) | 9.1 (-3.3, 21.4) |  | 0.215 |
| L–Threonine *Thr* | 12 | 116.0 (107.0, 125.0) | 106.0 (96.7, 116.0) | 9.5 (-2.5, 21.6) |  | 0.164 |
| Tryptophan *Trp* | 4 | 37.7 (32.3, 43.1) | 44.1 (38.7, 49.5) | -6.4 (-13.5, 0.7) |  | 0.088 |
| Tryptophan *Trp* | 8 | 38.5 (33.0, 44.1) | 42.1 (36.7, 47.5) | -3.6 (-10.8, 3.7) |  | 0.537 |
| Tryptophan *Trp* | 12 | 40.4 (35.2, 45.7) | 35.4 (30, 40.8) | 5.0 (-2.0, 12.0) |  | 0.234 |
| L–Tyrosine *Tyr* | 4 | 46.9 (43.2, 50.8) | 48.4 (44.7, 52.5) |  | 1.0 (0.9, 1.1) | 0.803 |
| L–Tyrosine *Tyr* | 8 | 54.6 (50.3, 59.3) | 50.5 (46.6, 54.8) |  | 1.1 (1.0, 1.2) | 0.198 |
| L–Tyrosine *Tyr* | 12 | 50.7 (46.9, 54.9) | 47.5 (43.8, 51.5) |  | 1.1 (1.0, 1.2) | 0.298 |
| L–Valine *Val* | 4 | 143.0 (125.0, 160.0) | 145 (127.0, 162.0) | -1.9 (-24.3, 20.5) |  | 0.996 |
| L–Valine *Val* | 8 | 174.0 (156.0, 192.0) | 141.0 (123.0, 158.0) | 33.2 (10.5, 56.0) |  | 0.001 |
| L–Valine *Val* | 12 | 172.0 (155.0, 189.0) | 150.0 (133.0, 168.0) | 21.3 (-0.9, 43.5) |  | 0.064 |

**(b) Red blood cell**

| Parameter (μM) | Test Phase Week | Test | Control | Difference (Test – Control) in Means | Fold Change (Test / Control) in Means | *P* value |
| --- | --- | --- | --- | --- | --- | --- |
| 1–Methyl–L–Histidine *1–mhis* | 4 | 11.4 (9.9, 13.1) | 13.4 (11.6, 15.3) |  | 0.9 (0.7, 1.0) | 0.098 |
| 1–Methyl–L–Histidine *1–mhis* | 8 | 11.5 (10.0, 13.2) | 13.4 (11.7, 15.4) |  | 0.9 (0.7, 1.0) | 0.090 |
| 1–Methyl–L–Histidine *1–mhis* | 12 | 8.8 (7.7, 10.1) | 10.9 (9.5, 12.5) |  | 0.8 (0.7, 1.0) | 0.015 |
| 3–Methyl–L–Histidine *3–mhis* | 4 | 5.6 (4.6, 6.7) | 5.9 (4.93, 7.08) |  | 0.9 (0.7, 1.2) | 0.927 |
| 3–Methyl–L–Histidine *3–mhis* | 8 | 5.7 (4.7, 6.8) | 5.9 (4.89, 7.03) |  | 1.0 (0.8, 1.2) | 0.974 |
| 3–Methyl–L–Histidine *3–mhis* | 12 | 3.2 (2.7, 3.9) | 3.8 (3.19, 4.58) |  | 0.9 (0.7, 1.1) | 0.251 |
| L–Alanine *Ala* | 4 | 375.0 (348.0, 404.0) | 368.0 (341.0, 397.0) |  | 1.0 (0.9, 1.1) | 0.927 |
| L–Alanine *Ala* | 8 | 411 (381.0, 443.0) | 414 (384.0, 446.0) |  | 1.0 (0.9, 1.1) | 0.995 |
| L–Alanine *Ala* | 12 | 396 (368.0, 427.0) | 380 (352.0, 410.0) |  | 1.0 (0.9, 1.2) | 0.576 |
| L–α–Amino–η Butyric acid *Aaba* | 4 | 10.2 (8.4, 12.2) | 8.55 (7.13, 10.3) |  | 1.2 (0.9, 1.5) | 0.229 |
| L–α–Amino–η Butyric acid *Aaba* | 8 | 7.57 (6.3, 9.1) | 6.2 (5.17, 7.4) |  | 1.2 (1.0, 1.6) | 0.128 |
| L–α–Amino–η Butyric acid *Aaba* | 12 | 10.7 (8.9, 12.8) | 10.3 (8.6, 12.4) |  | 1.0 (0.8, 1.3) | 0.981 |
| L–Arginine *Arg* | 4 | 82.3 (75.2, 90.2) | 89.5 (81.8, 97.8) |  | 0.9 (0.8, 1.0) | 0.226 |
| L–Arginine *Arg* | 8 | 95.8 (87.4, 105.0) | 97.9 (89.5, 107.0) |  | 1.0 (0.9, 1.1) | 0.954 |
| L–Arginine *Arg* | 12 | 97.7 (89.4, 107) | 95.5 (87.4, 104.0) |  | 1.0 (0.9, 1.2) | 0.951 |
| L–Asparagine *Asn* | 4 | 49.9 (45.8, 54.0) | 50.9 (46.9, 54.8) | -1.0 (-6.1, 4.2) |  | 0.956 |
| L–Asparagine *Asn* | 8 | 53.2 (49.2, 57.3) | 58.5 (54.5, 62.5) | -5.3 (-10.4, -0.1) |  | 0.044 |
| L–Asparagine *Asn* | 12 | 54.6 (50.6, 58.5) | 54.9 (51, 58.9) | -0.4 (-5.4, 4.7) |  | 0.998 |
| L–Aspartic Acid *Asp* | 4 | 1148.0 (1028.0, 1282.0) | 1159.0 (1039.0, 1292.0) |  | 1.0 (0.9, 1.1) | 0.997 |
| L–Aspartic Acid *Asp* | 8 | 1279.0 (1145.0, 1428.0) | 1225.0 (1098.0, 1366.0) |  | 1.0 (0.9, 1.2) | 0.811 |
| L–Aspartic Acid *Asp* | 12 | 931 (836.0, 1037.0) | 892.0 (800.0, 995.0) |  | 1.0 (0.9, 1.2) | 0.808 |
| L–Citrulline *Citr* | 4 | 6.09 (5.0, 7.43) | 6.6 (5.4, 8.0) |  | 0.9 (0.7, 1.2) | 0.831 |
| L–Citrulline *Citr* | 8 | 5.45 (4.47, 6.64) | 5.5 (4.6, 6.7) |  | 1.0 (0.8, 1.3) | 0.998 |
| L–Citrulline *Citr* | 12 | 5.29 (4.37, 6.4) | 4.7 (3.9, 5.7) |  | 1.1 (0.9, 1.5) | 0.529 |
| Glutamine *Gln* | 4 | 416 (388.0, 446.0) | 411.0 (383.0, 440.0) |  | 1.0 (0.9, 1.1) | 0.972 |
| Glutamine *Gln* | 8 | 410 (382.0, 439.0) | 425.0 (396.0, 455.0) |  | 1.0 (0.9, 1.1) | 0.657 |
| Glutamine *Gln* | 12 | 404 (378.0, 433.0) | 409.0 (382.0, 438.0) |  | 1.0 (0.9, 1.1) | 0.984 |
| L–Glutamic acid *Glu* | 4 | 47.8 (41.1, 55.6) | 47.7 (41.2, 55.2) |  | 1.0 (0.8, 1.2) | 1.00 |
| L–Glutamic acid *Glu* | 8 | 45.5 (39.2, 52.9) | 52.8 (45.6, 61.2) |  | 0.9 (0.7, 1.0)† | 0.169 |
| L–Glutamic acid *Glu* | 12 | 56.6 (49.0, 65.5) | 54.1 (46.7, 62.6) |  | 1.1 (0.9, 1.3)† | 0.909 |
| Glycine *Gly* | 4 | 295 (277.0, 312.0) | 246.0 (229, 263) | 48.7 (26.5, 70.8) |  | <0.001 |
| Glycine *Gly* | 8 | 280 (262.0, 297.0) | 259.0 (241, 276) | 21.3 (-0.9, 43.5) |  | 0.063 |
| Glycine *Gly* | 12 | 283 (266, 300) | 248 (231, 265) | 35.2 (13.3, 57.1) |  | <0.001 |
| L–Histidine *His* | 4 | 99.9 (93.9, 106) | 101 (95.7, 107) | -1.6 (-9.1, 6.0) |  | 0.938 |
| L–Histidine *His* | 8 | 107 (101, 113) | 116 (110, 122) | -9.1 (-16.6, -1.5) |  | 0.012 |
| L–Histidine *His* | 12 | 102 (96.2, 108) | 101 (95.2, 107) | 1.0 (-6.5, 8.4) |  | 0.984 |
| L–Isoleucine *Ile* | 4 | 48.3 (44.3, 52.8) | 49.7 (45.5, 54.2) |  | 1.0 (0.9, 1.1) | 0.899 |
| L–Isoleucine *Ile* | 8 | 57.5 (52.7, 62.8) | 50.1 (45.9, 54.7) |  | 1.2 (1.0, 1.3) | 0.012 |
| L–Isoleucine *Ile* | 12 | 57.9 (53.2, 63.1) | 52.6 (48.2, 57.4) |  | 1.1 (1.0, 1.2) | 0.11 |
| L–Leucine *Leu* | 4 | 117 (109, 126) | 117 (109, 125) |  | 1.0 (0.9, 1.1) | 1.00 |
| L–Leucine *Leu* | 8 | 142 (132, 153) | 144 (134, 155) |  | 1.0 (0.9, 1.1) | 0.975 |
| L–Leucine *Leu* | 12 | 150 (140, 160) | 141 (132, 151) |  | 1.1 (1.0, 1.2) | 0.317 |
| L–Lysine *Lys* | 4 | 137 (123, 150) | 149 (136, 162) | -12.4 (-29.6, 4.8) |  | 0.22 |
| L–Lysine *Lys* | 8 | 163 (150, 177) | 159 (146, 172) | 4.2 (-12.9, 21.3) |  | 0.899 |
| L–Lysine *Lys* | 12 | 153 (140, 166) | 145 (132, 159) | 7.7 (-9.3, 24.7) |  | 0.59 |
| Methionine *Met* | 4 | 39.4 (35.8, 43) | 40.5 (37, 44) | -1.1 (-5.6, 3.5) |  | 0.921 |
| Methionine *Met* | 8 | 36.2 (32.7, 39.8) | 43.5 (40, 47) | -7.3 (-11.8, -2.7) |  | >0.001 |
| Methionine *Met* | 12 | 39.9 (36.4, 43.4) | 41.2 (37.7, 44.7) | -1.3 (-5.8, 3.2) |  | 0.861 |
| L–Ornithine *Orn* | 4 | 4.28 (3.58, 5.12) | 4.48 (3.75, 5.34) |  | 1.0 (0.8, 1.2) | 0.945 |
| L–Ornithine *Orn* | 8 | 5.66 (4.74, 6.77) | 4.57 (3.83, 5.44) |  | 1.2 (1.0, 1.6) | 0.066 |
| L–Ornithine *Orn* | 12 | 5.11 (4.29, 6.08) | 4.71 (3.95, 5.61) |  | 1.1 (0.9, 1.4) | 0.733 |
| L–Phenylalanine *Phe* | 4 | 138 (127, 150) | 146 (134, 159) |  | 0.9 (0.8, 1.1) | 0.433 |
| L–Phenylalanine *Phe* | 8 | 157 (144, 171) | 159 (146, 173) |  | 1.0 (0.9, 1.1) | 0.971 |
| L–Phenylalanine *Phe* | 12 | 159 (146, 172) | 157 (144, 170) |  | 1.0 (0.9, 1.1) | 0.985 |
| L–Proline *Pro* | 4 | 100 (90.3, 111) | 99.7 (89.9, 110) | 0.7 (-12.1, 13.5) |  | 0.999 |
| L–Proline *Pro* | 8 | 100 (90.1, 110) | 119 (109, 129) | -18.8 (-31.6, -6.0) |  | 0.001 |
| L–Proline *Pro* | 12 | 95 (85.1, 105) | 93.8 (84, 104) | 1.2 (-11.4, 13.8) |  | 0.995 |
| L–Serine *Ser* | 4 | 54 (45.6, 64) | 52.7 (44.7, 62.2) |  | 1.0 (0.8, 1.3) | 0.989 |
| L–Serine *Ser* | 8 | 57 (48.1, 67,5) | 69.9 (59.3, 82.5) |  | 0.8 (0.7, 1.0) | 0.066 |
| L–Serine *Ser* | 12 | 67.4 (57.2, 79.4) | 62.9 (53.3, 74.2) |  | 1.1 (0.9, 1.3) | 0.81 |
| Taurine *Taur* | 4 | 74.1 (56.7, 96.7) | 65.7 (50.8, 85) |  | 1.1 (0.8, 1.6) | 0.779 |
| Taurine *Taur* | 8 | 63.5 (48.6, 82.8) | 72.6 (56.1, 93.9) |  | 0.9 (0.6, 1.2) | 0.711 |
| Taurine *Taur* | 12 | 87.6 (67.8, 113) | 77.8 (60.2, 101) |  | 1.1 (0.8, 1.6) | 0.774 |
| L–Threonine *Thr* | 4 | 48.1 (42.2, 54.9) | 48.5 (42.7, 55) |  | 1.0 (0.8, 1.2) | 0.999 |
| L–Threonine *Thr* | 8 | 46.4 (40.7, 52.8) | 54 (47.5, 61.2) |  | 0.9 (0.7, 1.0) | 0.082 |
| L–Threonine *Thr* | 12 | 53.2 (46.9, 60.4) | 51.3 (45.2, 58.2) |  | 1.0 (0.9, 1.2) | 0.93 |
| Tryptophan *Trp* | 4 | 20.1 (17.1, 23.1) | 20.9 (18, 23.9) | -0.8 (-4.7, 3.1) |  | 0.942 |
| Tryptophan *Trp* | 8 | 21.3 (18.2, 24.3) | 20 (17.1, 23) | 1.3 (-2.6, 5.1) |  | 0.815 |
| Tryptophan *Trp* | 12 | 10.9 (7.96, 13.8) | 11.4 (8.42, 14.3) | -0.5 (-4.3, 3.3) |  | 0.985 |
| L–Tyrosine *Tyr* | 4 | 192 (181, 204) | 203 (192, 214) | -10.6 (-25, 3.76) |  | 0.191 |
| L–Tyrosine *Tyr* | 8 | 195 (184, 206) | 204 (193, 215) | -9.2 (-23.6, 5.2) |  | 0.287 |
| L–Tyrosine *Tyr* | 12 | 188 (177, 199) | 198 (187, 209) | -9.8 (-24, 4.5) |  | 0.238 |
| L–Valine *Val* | 4 | 2.24 (2.21, 2.26) | 2.25 (2.22, 2.27) | -0.011 (-0.043, 0.021)‡ |  | 0.784 |
| L–Valine *Val* | 8 | 2.27 (2.24, 2.29) | 2.26 (2.24, 2.29 | 0.006 (-0.026, 0.038)‡ |  | 0.958 |
| L–Valine *Val* | 12 | 2.26 (2.23, 2.28) | 2.24 (2.22, 2.26) | 0.016 (-0.016, 0.047)‡ |  | 0.529 |

All values are means and brackets indicate 95% confidence intervals of the mean (*P* ≤ 0.05). Brackets indicate 95% confidence intervals of the mean. ‡ indicates that a linear model with week, diet and their interaction without a random effect was fit to the variable L-Valine (*Val*).
