# Supplementary material for: Glycine supplementation can partially restore oxidative stress-associated glutathione deficiency in ageing cats
Source: Br J Nutr. 2024 Feb 29;131(12):1947–61. doi: 10.1017/S0007114524000370 (PMC11361917; doi:10.1017/S0007114524000370)
Supplement: Ruparell et al. supplementary material 6 — Ruparell et al. supplementary material [file S0007114524000370sup006.docx]

**Supplementary Table 4. Biochemistry parameter values for the GLY feeding study test (supplemented) and control (unsupplemented) senior cats.**

| Parameter | Test Phase Week | Test | Control | Difference in means (Test – Control) | *P* value |
| --- | --- | --- | --- | --- | --- |
| Glucose (mg/dL) | 4 | 74.1 (66.2, 82.1) | 65.3 (57.4, 73.3) | 8.82 (-1.42, 19.1) | 0.114 |
| Glucose (mg/dL) | 8 | 74.7 (66.5, 82.9) | 69.2 (60.4, 78.0) | 5.53 (-5.4, 16.5) | 0.536 |
| Glucose (mg/dL) | 12 | 72.3 (64.2, 80.3) | 70.5 (62.2, 78.7) | 1.8 (-8.66, 12.3) | 0.967 |
| SDMA (μg/dL) | 4 | 11.5 (10.1, 13.0) | 11.8 (10.3, 13.3) | -0.265 (-2.19, 1.66) | 0.98 |
| SDMA (μg/dL) | 8 | 14.5 (13.0, 16.1) | 11.8 (10.2, 13.3) | 2.76 (0.745, 4.78) | 0.004 |
| SDMA (μg/dL) | 12 | 13.4 (11.9, 14.9) | 10.7 (9.21, 12.3) | 2.62 (0.66, 4.58) | 0.005 |
| Creatinine (mg/dL) | 4 | 1.49 (1.4, 1.59) | 1.42 (1.32, 1.51) | 0.0771 (-0.0446, 0.199) | 0.327 |
| Creatinine (mg/dL) | 8 | 1.41 (1.31, 1.5) | 1.42 (1.32 (1.52) | -0.0121 (-0.138, 0.114) | 0.993 |
| Creatinine (mg/dL) | 12 | 1.53 (1.44, 1.62) | 1.45 (1.35, 1.55) | 0.0821 (-0.0407, 0.205) | 0.284 |
| BUN (mg/dL) | 4 | 22.0 (20.8, 23.2) | 21.0 (19.7, 22.2) | 1.02 (-0.65, 2.69) | 0.321 |
| BUN (mg/dL) | 8 | 22.2 (20.9, 23.4) | 20.8 (19.5, 22.1) | 1.38 (-0.32, 3.08) | 0.135 |
| BUN (mg/dL) | 12 | 21.3 (20.1, 22.5) | 19.4 (18.1, 20.7) | 1.88 (0.189, 3.57) | 0.025 |
| BUN:Crea | 4 | 14.9 (13.8, 16.1) | 14.9 (13.8, 16.1) | -0.000283 (-1.48, 1.48) | 1.00 |
| BUN:Crea | 8 | 16.1 (14.9, 17.3) | 14.8 (13.6, 16.1) | 1.26 (-0.297, 2.81) | 0.150 |
| BUN:Crea | 12 | 13.9 (12.8, 15.1) | 13.5 (12.3, 14.7) | 0.467 (-1.05, 1.98) | 0.838 |
| Phosphorous (mg/dL) | 4 | 4.26 (3.98, 4.53) | 4.17 (3.88, 4.46) | 0.0889 (-0.272, 0.45) | 0.893 |
| Phosphorous (mg/dL) | 8 | 3.98 (3.71, 4.26) | 4.50 (4.20, 4.81) | -0.519 (-0.893, -0.144) | 0.003 |
| Phosphorous (mg/dL) | 12 | 3.88 (3.61, 4.15) | 3.88 (3.59, 4.17) | 0.00112 (-0.36, 0.362) | 1.00 |
| Calcium (mg/dL) | 4 | 9.32 (9.1, 9.54) | 9.26 (9.03, 9.48) | 0.0623 (-0.229, 0.354) | 0.932 |
| Calcium (mg/dL) | 8 | 9.1 (8.88, 9.32) | 9.1 (8.85, 9.35) | 0.00382 (-0.302, 0.31) | 1.00 |
| Calcium (mg/dL) | 12 | 8.76 (8.54, 8.97) | 8.73 (8.5, 8.96) | 0.026 (-0.267, 0.319) | 0.994 |
| Sodium (mM/L) | 4 | 151 (149, 152) | 150 (149, 1.52) | 0.467 (-1.48, 2.42) | 0.918 |
| Sodium (mM/L) | 8 | 150 (148, 151) | 149 (147, 150) | 1.07 (-1.01, 3.15) | 0.521 |
| Sodium (mM/L) | 12 | 150 (148, 152) | 151 (149, 153) | -0.984 (-3, 1.03) | 0.564 |
| Potassium (mM/L) | 4 | 4.52 (4.33, 4.7) | 4.59 (4.41, 4.77) | -0.0716 (-0.308, 0.165) | 0.832 |
| Potassium (mM/L) | 8 | 4.4 (4.21, 4.59) | 4.64 (4.44, 4.83) | -0.238 (-0.487, 0.011) | 0.065 |
| Potassium (mM/L) | 12 | 4.29 (4.11, 4.48) | 4.38 (4.19, 4.57) | -0.0845 (-0.326, 0.157) | 0.765 |
| Na:K | 4 | 33.5 (32.2, 34.8) | 33.1 (31.8, 34.5) | 0.363 (-1.36, 2.09) | 0.932 |
| Na:K | 8 | 34.2 (32.8, 35.6) | 32.3 (30.9, 33.7) | 1.92 (0.113, 3.73) | 0.034 |
| Na:K | 12 | 35.1 (33.8, 36.5) | 34.7 (33.3, 36.1) | 0.431 (-1.32, 2.18) | 0.898 |
| Chloride (mM/L) | 4 | 118 (116, 119) | 117 (115, 118) | 0.885 (-0.975, 2.75) | 0.586 |
| Chloride (mM/L) | 8 | 117 (115, 118) | 116 (114, 117) | 1.14 (-0.853, 3.12) | 0.432 |
| Chloride (mM/L) | 12 | 117 (115, 118) | 118 (117, 120) | -1.31 (-3.23, 0.615) | 0.28 |
| TCO_2_ (mM/L) | 4 | 18 (17, 19.1) | 18.4 (17.3, 19.4) | -0.337 (-1.69, 1.01) | 0.897 |
| TCO_2_ (mM/L) | 8 | 16.4 (15.3, 17.5) | 16.9 (15.8, 18.0) | -0.522 (-1.92, 0.881) | 0.731 |
| TCO_2_ (mM/L) | 12 | 15.5 (14.4, 16.5) | 16.2 (15.1 (17.3) | -0.715 (-2.09, 0.656) | 0.486 |
| Anion Gap (mM/L) | 4 | 19.8 (18.3, 21.2) | 19.9 (18.4, 21.4) | -0.146 (-2.04, 1.74) | 0.996 |
| Anion Gap (mM/L) | 8 | 20.7 (19.2, 22.3) | 21.1 (19.5, 22.7) | -0.357 (-2.35, 1.64) | 0.958 |
| Anion Gap (mM/L) | 12 | 21.8 (20.2, 23.3) | 21 (19.5, 22.5) | 0.748 (-1.19, 2.69) | 0.71 |
| Total Protein (g/dL) | 4 | 7.35 (7.12, 7.57) | 7.27 (7.05, 7.5) | 0.0735 (-0.215, 0.362) | 0.88 |
| Total Protein (g/dL) | 8 | 7.48 (7.25, 7.71) | 7.46 (7.23, 7.7) | 0.0108 (-0.289, 0.31) | 1.00 |
| Total Protein (g/dL) | 12 | 7.02 (6.79, 7.24) | 6.95 (6.71, 7.18) | 0.0705 (-0.221, 0.362) | 0.895 |
| Albumin (g/dL) | 4 | 2.8 (2.7, 2.89) | 2.81 (2.71, 2.91) | -0.017 (-0.143, 0.109) | 0.983 |
| Albumin (g/dL) | 8 | 2.81 (2.71, 2.91) | 2.81 (2.71, 2.92) | -0.00333 (-0.135, 0.128) | 1.00 |
| Albumin (g/dL) | 12 | 2.75 (2.65, 2.85) | 2.68 (2.58, 2.78) | 0.0746 (-0.0527, 0.202) | 0.402 |
| Globulin (g/dL) | 4 | 4.55 (4.36, 4.75) | 4.46 (4.26, 4.65) | 0.0985 (-0.149, 0.346) | 0.656 |
| Globulin (g/dL) | 8 | 4.67 (4.48, 4.87) | 4.65 (4.44, 4.85) | 0.0291 (-0.226, 0.284) | 0.986 |
| Globulin (g/dL) | 12 | 4.27 (4.08, 4.46) | 4.26 (4.06, 4.46) | 0.0064 (-0.243, 0.256) | 1.00 |
| Albumin:Globulin | 4 | 0.624 (0.584, 0.665) | 0.64 (0.599, 0.681) | -0.0161 (-0.0682, 0.0359) | 0.814 |
| Albumin:Globulin | 8 | 0.612 (0.571, 0.654) | 0.607 (0.565, 0.65) | 0.00472 (-0.0492, 0.0586) | 0.994 |
| Albumin:Globulin | 12 | 0.66 (0.62, 0.701) | 0.631 (0.589 (0.673) | 0.0289 (-0.0238, 0.0815) | 0.435 |
| ALT (U/L) | 4 | 45.6 (40.1, 51.9) | 42.9 (37.7, 48.9) | 1.06 (0.901, 1.25) | 0.669 |
| ALT (U/L) | 8 | 44.7 (39.3, 51) | 41.7 (36.4, 47.7) | 1.07 (0.908, 1.27) | 0.579 |
| ALT (U/L) | 12 | 48.8 (42.9, 55.5) | 41.7 (36.5, 47.6) | 1.17 (0.992, 1.38) | 0.066 |
| AST (U/L) | 4 | 24.6 (21.6, 28.1) | 22.3 (19.6, 25.5) | 1.1 (0.932, 1.31) | 0.39 |
| AST (U/L) | 8 | 27.2 (23.8, 31.1) | 23.7 (20.6, 27.2) | 1.15 (0.965, 1.37) | 0.154 |
| AST (U/L) | 12 | 24.3 (31.3, 27.7) | 22.5 (19.6, 25.7) | 1.08 (0.91, 1.28) | 0.597 |
| ALP (U/L) | 4 | 28.7 (26.3, 31.1) | 27.7 (25.4, 30.1) | 0.977 (-2.09, 4.05) | 0.815 |
| ALP (U/L) | 8 | 31.3 (28.9, 33.8) | 30.4 (27.9, 32.9) | 0.935 (-2.27, 4.14) | 0.850 |
| ALP (U/L) | 12 | 29.6 (27.3, 32) | 29.5 (27, 31.9) | 0.194 (-2.92, 3.31) | 0.998 |
| Cholesterol (mg/dL) | 4 | 141 (132, 150) | 155 (146, 165) | -14.3 (-26.1, -2.54) | 0.011 |
| Cholesterol (mg/dL) | 8 | 145 (135, 154) | 167 (157, 177) | -22.2 (-34.5, -9.88) | <0.001 |
| Cholesterol (mg/dL) | 12 | 153 (144, 162) | 142 (133, 152) | 10.3 (-1.62, 22.3) | 0.111 |
| Creatine Kinase (U/L) | 4 | 191 (145, 252) | 146 (110, 193) | 1-31 (0.92, 1.87) | 0.172 |
| Creatine Kinase (U/L) | 8 | 211 (159, 280) | 142 (106, 190) | 1.49 (1.03 (2.15) | 0.029 |
| Creatine Kinase (U/L) | 12 | 169 (128, 223) | 146 (110, 194) | 1.16 (0.809, 1.66) | 0.64 |

All values are means and brackets indicate 95% confidence intervals of the mean (*P* ≤0.05). ALT, alanine aminotransferase; ALP, alkaline phosphatase; AST, aspartate aminotransferase; BUN, blood urea nitrogen; SDMA, symmetric dimethylarginine; TCO_2_, bicarbonate.
