# Supplementary material for: Glycine supplementation can partially restore oxidative stress-associated glutathione deficiency in ageing cats
Source: Br J Nutr. 2024 Feb 29;131(12):1947–61. doi: 10.1017/S0007114524000370 (PMC11361917; doi:10.1017/S0007114524000370)
Supplement: Ruparell et al. supplementary material 7 — Ruparell et al. supplementary material [file S0007114524000370sup007.docx]

**Supplementary Table 5. Haematology parameter values for the GLY feeding study test (supplemented) and control (unsupplemented) senior cats.**

| Parameter | Test Phase Week | Test | Control | Difference in means (Test – Control) | *P* value |
| --- | --- | --- | --- | --- | --- |
| RBC (M/μL) | 4 | 8.44 (7.75, 9.02) | 8.58 (8.01, 9.15) | -0.143 (-0.88, 0.594) | 0.946 |
| RBC (M/μL) | 8 | 9.27 (8.67, 9.87) | 9.37 (8.79, 9.95) | -0.0996 (-0.855, 0.656) | 0.982 |
| RBC (M/μL) | 12 | 8.91 (8.33, 9.49) | 9.23 (8.66, 9.80) | -0.322 (-1.06, 0.416) | 0.620 |
| Haematocrit (%) | 4 | 36.6 (34.3, 39.0) | 37.2 (35.0, 39.5) | -0.614 (-3.57, 2.34) | 0.940 |
| Haematocrit (%) | 8 | 39.4 (37.0, 41.9) | 40.4 (38.1, 42.8) | -0.981 (-4.03, 2.06) | 0.812 |
| Haematocrit (%) | 12 | 38.0 (35.7, 40.3) | 38.9 (36.7, 41.2) | -0.935 (-3.9, 2.03) | 0.821 |
| Haemoglobin (g/dL) | 4 | 11.5 (10.7, 12.2) | 11.8 (11.1 12.5) | -0.332 (-1.25, 0.591) | 0.758 |
| Haemoglobin (g/dL) | 8 | 12.5 (11.7, 13.2) | 12.6 (11.9, 13.4) | -0.177 (-1.13, 0.773) | 0.955 |
| Haemoglobin (g/dL) | 12 | 11.9 (11.1, 12.6) | 12.4 (11.7, 13.1) | -0.565 (-1.49, 0.36) | 0.360 |
| MCV (fL) | 4 | 43.7 (43.1, 44.4) | 43.5 (42.9, 44.1) | 1.01 (0.987, 1.03) | 0.829 |
| MCV (fL) | 8 | 42.8 (42.2, 43.5) | 43.0 (42.3, 43.6) | 0.996 (0.977, 1.02) | 0.956 |
| MCV (fL) | 12 | 43.0 (42.4, 43.7) | 42.1 (41.5, 42.7) | 1.02 (1.00, 1.04) | 0.021 |
| MCH (pg) | 4 | 13.7 (13.5, 13.9) | 13.8 (13.6, 14.0) | -0.0822 (-0.318, 0.154) | 0.739 |
| MCH (pg) | 8 | 13.5 (13.4, 13.7) | 13.4 (13.3, 13.6) | 0.0986 (-0.142, 0.339) | 0.637 |
| MCH (pg) | 12 | 13.4 (13.3, 13.6) | 13.5 (13.3, 13.7) | -0.0515 (-0.287, 0.184) | 0.914 |
| MCHC (g/dL) | 4 | 31.3 (30.7, 31.8) | 31.7 (31.2 32.2) | -0.421 (-1.13, 0.292) | 0.401 |
| MCHC (g/dL) | 8 | 31.6 (31.0, 32.1) | 31.3 (30.7, 31.8) | 0.278 (-0.457, 1.01) | 0.743 |
| MCHC (g/dL) | 12 | 31.2 (30.7, 31.8) | 32.0 (31.4, 32.5) | -0.746 (-1.46, -0.0327) | 0.037 |
| % Reticulocyte (%) | 4 | 0.124 (0.0842, 0.182) | 0.114 (0.0782, 0.165) | 1.09 (0.667, 1.78) | 0.967 |
| % Reticulocyte (%) | 8 | 0.121 (0.0812, 0.181) | 0.131 (0.0895, 0.193) | 0.922 (0.557, 1.53) | 0.973 |
| % Reticulocyte (%) | 12 | 0.107 (0.0725, 0.157) | 0.0826 (0.0568, 0.12) | 1.29 (0.791, 2.11) | 0.510 |
| Reticulocytes (K/μL) | 4 | 10.3 (6.58, 14) | 10.3 (6.7, 13.9) | -0.0114 (-4.71, 4.69) | 1.00 |
| Reticulocytes (K/μL) | 8 | 10.8 (6.99, 14.6) | 12.2 (8.52, 15.9) | -1.4 (-6.22, 3.42) | 0.864 |
| Reticulocytes (K/μL) | 12 | 11.4 (7.66, 15.1) | 9.7 (6.11, 13.3) | 1.66 (-3.04, 6.36) | 0.781 |
| Reticulocyte Haemoglobin (pg) | 4 | 15.9 (15.2, 16.7) | 15.3 (14.6, 16.0) | 1.04 (0.984, 1.11) | 0.227 |
| Reticulocyte Haemoglobin (pg) | 8 | 14.9 (14.2, 15.6) | 15.0 (14.4, 15.8) | 0.991 (0.932, 1.05) | 0.974 |
| Reticulocyte Haemoglobin (pg) | 12 | 15.1 (14.4, 15.8) | 14.9 (14.2, 15.6) | 1.01 (0.954, 1.08) | 0.920 |
| WBC (K/μL) | 4 | 15.3 (12.8, 18.3) | 13.3 (11.1, 15.8) | 1.15 (0.92, 1.45) | 0.335 |
| WBC (K/μL) | 8 | 12.6 (10.5, 15.1) | 12.0 (10.0, 14.4) | 1.05 (0.83, 1.32) | 0.946 |
| WBC (K/μL) | 12 | 11.7 (9.79, 14.0) | 11.5 (9.67, 13.7) | 1.02 (0.811, 1.28) | 0.997 |
| % Neutrophils (%) | 4 | 65.4 (59.8, 71.0) | 63.9 (58.4, 69.4) | 1.5 (-5.61, 8.62) | 0.937 |
| % Neutrophils (%) | 8 | 71.2 (65.4, 77.0) | 66.5 (60.9, 72.2) | 4.7 (-2.62, 12.0) | 0.320 |
| % Neutrophils (%) | 12 | 65.7 (60.1, 71.3) | 69.7 (64.2, 75.2) | -3.99 (-11.1, 3.14) | 0.473 |
| % Lymphocytes (%) | 4 | 16.5 (12.5, 20.5) | 17.2 (13.4, 21.1) | -0.739 (-5.76, 4.28) | 0.979 |
| % Lymphocytes (%) | 8 | 19.4 (15.3, 23.5) | 21.9 (18.0, 25.9) | -2.53 (-7.71, 2.65) | 0.565 |
| % Lymphocytes (%) | 12 | 21.9 (17.9, 25.9) | 18.2 (14.3, 22.1) | 3.7 (-1.32, 8.73) | 0.215 |
| % Monocytes (%) | 4 | 4.23 (3.35, 5.1) | 4.14 (3.29, 4.99) | 0.0856 (-1.03, 1.2) | 0.996 |
| % Monocytes (%) | 8 | 3.57 (2.67, 4.47) | 4.16 (3.29, 5.04) | -0.593 (-1.74, 0.557) | 0.498 |
| % Monocytes (%) | 12 | 4.08 (3.2, 4.95) | 3.76 (2.91, 4.62) | 0.315 (-0.802, 1.43) | 0.861 |
| % Eosinophils (%) | 4 | 10.3 (7.54, 14) | 11.8 (8.77, 15.9) | 0.868 (0.588, 12.8) | 0.765 |
| % Eosinophils (%) | 8 | 5.34 (3.89, 7.33) | 6.54 (4.81, 8.89) | 0.817 (0.547, 1.22) | 0.536 |
| % Eosinophils (%) | 12 | 7.73 (5.69, 10.5) | 7.61 (5.65, 10.3) | 1.02 (0.688, 1.5) | 1.00 |
| % Basophils (%) | 4 | 0.255 (0.153, 0.426) | 0.108 (0.0654, 0.177) | 2.37 (1.24, 4.54) | 0.004 |
| % Basophils (%) | 8 | 0.217 (0.128, 0.368) | 0.245 (0.147, 0.409) | 0.884 (0.453, 1.72) | 0.960 |
| % Basophils (%) | 12 | 0.193 (0.116, 0.323) | 0.204 (0.124, 0.335) | 0.949 (0.497, 1.81) | 0.996 |
| Neutrophils (K/μL) | 4 | 9.83 (7.96, 12.1) | 8.37 (6.82, 10.3) | 1.17 (0.9, 1.53) | 0.367 |
| Neutrophils (K/μL) | 8 | 8.93 (7.19, 11.1) | 7.92 (6.42, 9.78) | 1.13 (0.857, 1.48) | 0.629 |
| Neutrophils (K/μL) | 12 | 7.62 (6.17, 9.41) | 7.97 (6.49, 9.79) | 0.956 (0.732, 1.25) | 0.964 |
| Lymphocytes (K/μL) | 4 | 2.32 (1.94, 2.77) | 2.18 (1.83, 2.59) | 1.06 (0.849, 1.33) | 0.881 |
| Lymphocytes (K/μL) | 8 | 2.18 (1.81, 2.61) | 2.54 (2.12, 3.03) | 0.859 (0.682, 1.08) | 0.300 |
| Lymphocytes (K/μL) | 12 | 2.38 (2.00, 2.85) | 2.05 (1.72, 2.43) | 1.17 (0.931, 1.46) | 0.271 |
| Monocytes (K/μL) | 4 | 0.579 (0.437, 0.767) | 0.525 (0.398, 0.691) | 1.1 (0.77, 1.58) | 0.873 |
| Monocytes (K/μL) | 8 | 0.416 (0.310, 0.556) | 0.495 (0.373, 0.656) | 0.84 (0.58, 1.22) | 0.574 |
| Monocytes (K/μL) | 12 | 0.441 (0.332, 0.585) | 0.417 (0.316, 0.549) | 1.06 (0.737, 1.52) | 0.973 |
| Eosinphils (K/μL) | 4 | 1.54 (1.08, 2.21) | 1.58 (1.11, 2.25) | 0.976 (0.618, 1.54) | 0.999 |
| Eosinphils (K/μL) | 8 | 0.663 (0.457, 0.963) | 0.797 (0.556, 1.14) | 0.832 (0.519, 1.33) | 0.723 |
| Eosinphils (K/μL) | 12 | 0.896 (0.624, 1.29) | 0.885 (0.624, 1.26) | 1.01 (0.641, 1.6) | 1.00 |
| Basophils (K/μL) | 4 | 0.0359 (0.0198, 0.0651) | 0.0126 (0.00706, 0.0223) | 2.86 (1.35, 6.07) | 0.003 |
| Basophils (K/μL) | 8 | 0.0268 (0.0145, 0.0495) | 0.029 (0.016, 0.0525) | 0.925 (0.427, 2.01) | 0.993 |
| Basophils (K/μL) | 12 | 0.0223 (0.0123, 0.0404) | 0.0229 (0.0129, 0.0408) | 0.971 (0.485, 2.06) | 1.00 |
| Platelets (K/μL) | 4 | 413 (348, 477) | 373 (310, 436) | 39.5 (-42.3, 121) | 0.566 |
| Platelets (K/μL) | 8 | 448 (381, 515) | 455 (391, 520) | -7.4 (-91.6, 76.8) | 0.995 |
| Platelets (K/μL) | 12 | 409 (344, 474) | 398 (335, 461) | 11.2 (-70.7, 93) | 0.982 |

All values are means and brackets indicate 95% confidence intervals of the mean (*P* ≤ 0.05). RBC, red blood cell; MCH, mean corpuscular haemoglobin; MCHC, mean corpuscular haemoglobin concentration; MCV, mean cell volume; WBC, white blood cell.
